# Tunable Multicolor Fluorescence of Perovskite-Based Composites for Optical Steganography and Light-Emitting Devices

**DOI:** 10.34133/2022/9896548

**Published:** 2022-09-13

**Authors:** Kewei Ma, Qingfeng Gui, Cihui Liu, Yunyi Yang, Fangjian Xing, Yunsong Di, Xiaoming Wen, Baohua Jia, Zhixing Gan

**Affiliations:** ^1^Center for Future Optoelectronic Functional Materials, School of Computer and Electronic Information/School of Artificial Intelligence, Nanjing Normal University, Nanjing 210023, China; ^2^College of Naval Architecture and Ocean Engineering, Jiangsu Maritime Institute, Nanjing 211170, China; ^3^Centre for Translational Atomaterials, School of Science, Swinburne University of Technology, John Street Hawthorn, VIC 3122, Australia; ^4^School of Science, RMIT University, Melbourne, 3000 VIC, Australia; ^5^College of Materials Science and Engineering, Qingdao University of Science and Technology, Qingdao 266042, China

## Abstract

Multicolor fluorescence of mixed halide perovskites enormously enables their applications in photonics and optoelectronics. However, it remains an arduous task to obtain multicolor emissions from perovskites containing single halogen to avoid phase segregation. Herein, a fluorescent composite containing Eu-based metal-organic frameworks (MOFs), 0D Cs_4_PbBr_6_, and 3D CsPbBr_3_ is synthesized. Under excitations at 365 nm and 254 nm, the pristine composite emits blue (B) and red (R) fluorescence, which are ascribed to radiative defects within Cs_4_PbBr_6_ and ^5^D_0_→^7^F_J_ transitions of Eu^3+^, respectively. Interestingly, after light soaking in the ambient environment, the blue fluorescence gradually converts into green (G) emission due to the defect repairing and 0D-3D phase conversion. This permanent and unique photochromic effect enables anticounterfeiting and microsteganography with increased security through a micropatterning technique. Moreover, the RGB luminescence is highly stable after encapsulation by a transparent polymer layer. Thus, trichromatic light-emitting modules are fabricated by using the fluorescent composites as color-converting layers, which almost fully cover the standard color gamut. Therefore, this work innovates a strategy for construction of tunable multicolor luminescence by manipulating the radiative defects and structural dimensionality.

## 1. Introduction

During the past years, there has been an unprecedentedly rapid development of lead halide perovskites, which is powered by their outstanding optoelectronic properties and extensive applications in solar cells, light-emitting diodes (LEDs), transistors, lasers, and scintillators [1–8]. The outstanding external quantum efficiency (EQE) over 20% and tunable wavelength from 400 to 740 nm by adjusting the halide compositions are the superior advantages of perovskite LEDs (PeLEDs) [9–12]. Besides, the controllable multicolor fluorescence also promotes the development of anticounterfeiting labels [13–15]. For instance, perovskite quantum dots (PQDs) with controlled halide compositions were embedded into pretreated polymer gel to prepare a printing ink [15]. Multicolor fluorescence patterns and two-dimensional codes were printed for polychromatic anticounterfeiting applications with enhanced safety. On the other hand, micropatterning to modulate fluorescence color enables additional feature/information to be encoded, significantly enhancing data security [16, 17]. Zhou et al. demonstrated the fabrication of various multicolor micropatterns by femtosecond direct laser writing (DLW) on gradient mixed halide perovskites, which paves the way for micro steganography and anticounterfeiting. Therefore, multicolor fluorescence exhibits a great prospect for broad applications in photonics and optoelectronics.

Multicolor fluorescence can be readily obtained from mixed halide perovskites by adjusting the halide compositions. However, the mixed halide perovskites suffer from phase segregation, resulting in poor stability [17–23]. Therefore, it is still urgent to develop single-halide perovskites with multicolor emissions to avoid the intrinsic instability caused by phase segregation. Introducing more emissive centers by ion doping is a potential strategy to acquire multicolor emissions in the single-halide perovskites [24–26]. In our previous work, Mn^2+^ doped CsPbCl_3_ (Mn:CsPbCl_3_) perovskite nanocrystals (PNCs) were prepared to launch a new emission at 600 nm in addition to the excitonic emission of CsPbCl_3_ at ca. 405 nm. Through anion exchange with CsPbBr_3_ PNCs, fluorescence covering from blue to orange are obtained [27]. Although iodine-based perovskite is no longer needed, the Mn:CsPb(Cl/Br)_3_ PNCs still cannot be free from phase segregation, and the pure red emission is missing.

Doping of lanthanide elements is a recognized approach to modulate the fluorescence of perovskites [28–30]. Zeng et al. [31] codoped Yb^3+^/Er^3+^/Bi^3+^ into perovskite single crystal. The products showed yellow, warm white, and green fluorescence under different excitation lights, which orients a direction for the development of ecofriendly and high-quality anticounterfeiting technology. Generally, intraconfigurational f–f transitions of lanthanides are strictly spin and parity forbidden, and the fluorescence is quite weak due to its extremely low absorption coefficient. Only when the 4f levels are coupled with orbitals having opposite-parity wavefunctions, such as 5d orbitals, the selection rules are partially allowed by spin–orbit coupling [32, 33]. Therefore, crystal-field perturbations via coupling between organic ligands and lanthanide ions have become an important way to enhance luminous efficiency [32–37]. Cortecchia et al. [38] synthesized an Eu^3+^-tetrakis *β*-diketonate metal-organic frameworks (MOFs) to dope 2D layered perovskites. And they demonstrated the sensitization by tetrakis *β*-diketonate complex endowing an appropriate coordination geometry and energetic landscape for the energy transfer to Eu^3+^, leading to a nearly 30-fold improvement in luminescence yield. Although lanthanide doping can introduce an additional emission color, it is still a great challenge to obtain multicolor fluorescence that covers the entire visible range, hindering the applications in anticounterfeiting, full color LEDs, and displays.

In this work, Eu-MOFs and radiative defects are jointly introduced to endow single-halide perovskites with multicolor fluorescence. Fluorescent composites containing Eu-benzenetricarboxylic (Eu-BTC) MOFs, 0D Cs_4_PbBr_6_, and 3D CsPbBr_3_ perovskites were prepared by a simple solution method. The as-prepared Eu-MOFs/perovskites composites emitted blue and red fluorescence when excited at 365 nm and 254 nm, respectively. Interestingly, under continuous light soaking, the blue fluorescence gradually disappeared, while the green fluorescence quickly grew. Thus, a fluorescent micropatterning technique was developed based on this interesting photochromic effect. Moreover, the trichromatic fluorescence that nearly coves the entire visible range can be obtained from the pristine and light-treated Eu-MOFs/perovskites composites. Therefore, efficient trichromatic LED modules were fabricated by using the Eu-MOFs/perovskites composites as color-converting layers. The long-term stability of the color-converting layers was demonstrated.

## 2. Results and Discussion

### 2.1. Structures of the Eu-MOFs/Perovskites Composites

**Figure 1:**
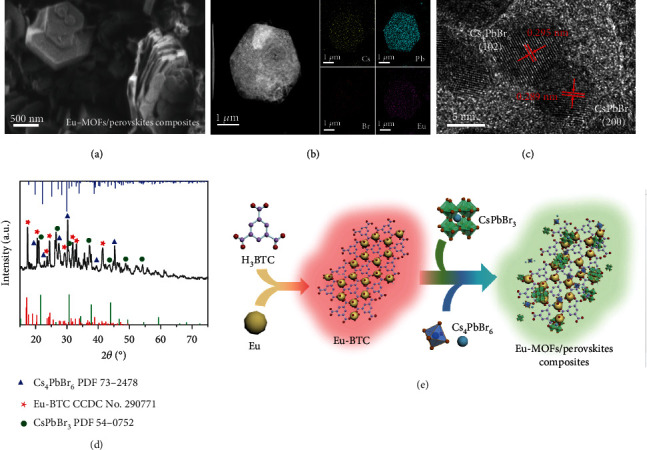
Structural characterizations. (a–d) SEM image (a), EDS mappings (b), HRTEM image (c), and XRD pattern (d) of the Eu-MOFs/perovskites composites. (e) Schematic diagram illustrating the formation of Eu-MOFs/perovskites composites.

Eu-MOFs/perovskites composites were prepared by a simple solvent method under mild reaction conditions at room temperature, as detailed in the experimental section. Scanning electron microscopic (SEM) images of the Eu-MOFs/perovskites composites are shown in Figure 1(a) and Figure S1. Most of the Eu-MOFs/perovskites composites show morphologies of lamellar flakes with typical hexagonal shapes, which may benefit from the framework played by the MOFs. The thickness of a single flake is about 50 nm to 100 nm with a relatively nonuniform distribution. And the edge length is about 0.5 to 2 *μ*m. The compositional distribution is investigated by energy dispersive X-ray spectroscopy (EDS) equipped on the transmission electron microscope (TEM) (Figure 1(b) and Figure S2), which show uniform distributions of Cs, Pb, Br, and Eu. High-resolution transmission electron microscopic (HRTEM) results reveal the coexistence of 0D Cs_4_PbBr_6_ and 3D CsPbBr_3_ nanocrystals on the hexagonal flakes (Figure 1(c) and Figure S3). Moreover, the presence of Eu in the form of Eu^3+^ is confirmed by the X-ray photoelectron spectroscopy (XPS) results (Figure S4).

Figure 1(d) presents X-ray diffraction (XRD) pattern of the Eu-MOFs/perovskites composites. The diffraction peaks related to CsPbBr_3_ and Eu-BTC are marked according to PDF#54-0752 and CCDC No. 290771, respectively. The obvious peaks at 21.6°, 30.6°, and 37.8° are attributed to the (110), (200), and (211) crystal planes of CsPbBr_3_. In addition, due to the mild reaction conditions, 0D perovskite (Cs_4_PbBr_6_, PDF#73-2478) also coexists in the product in addition to CsPbBr_3_ and Eu-BTC. The diffraction peaks at 22.4° and 30.2° are ascribed to the (300) and (214) crystal facets of the rhombohedra Cs_4_PbBr_6_. The diversity of structural dimensionality affords the opportunity for multicolor emission. According to the structural analysis, the structure of the Eu-MOFs/perovskites composites is illustrated in Figure 1(e). Eu-BTC self-assemble into a flake structure during the reaction, which is encapsulated by perovskite nanocrystals.

### 2.2. Photoluminescence of the Eu-MOFs/Perovskites Composites

**Figure 2:**
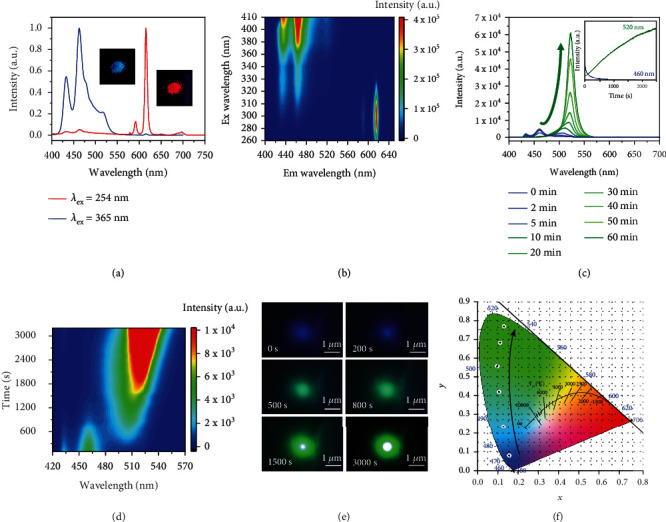
PL and light soaking induced PL variations. (a) PL spectra of the pristine Eu-MOFs/perovskites composites excited at 365 nm and 254 nm. Insets: the corresponding fluorescent photos. (b) Pseudocolor excitation-emission contour mapping. (c) PL spectra of the Eu-MOFs/perovskites composites during UV light soaking. Inset: variations of the PL intensities at 460 nm (blue line) and 520 nm (green line) with irradiation time. (d) Pseudocolor PL contour mapping during UV light soaking. (e) Fluorescence microscopic images acquired after different irradiation times. (f) CIE coordinates of the PL during light soaking. The excitation wavelength is 365 nm for the results shown in (b–e).

Photoluminescence (PL) spectra of the Eu-MOFs/perovskites composites are shown in Figure 2(a). When the excitation wavelength is 365 nm, the PL spectrum mainly contains four peaks at 433 nm, 460 nm, 520 nm, and 618 nm, respectively. The two blue peaks at 433 nm and 460 nm are significantly stronger than the other two. In particular, the 618 nm peak is extremely tiny. Therefore, under excitation at 365 nm, the fluorescence color is blue. When the excitation wavelength is changed to 254 nm, the PL peaks at 433 nm, 460 nm, and 520 nm almost vanish. Meanwhile, other emission peaks at 579 nm, 591 nm, 648 nm, and 697 nm appear. It is worth noting that the 618 nm PL peak becomes the strongest among all the bands. Consequently, the fluorescence color turns to red. The excitation-emission mapping is shown in Figure 2(b). With the increment of excitation wavelength, the dominant PL peak position jumps from 618 nm to 460 nm. Therefore, blue (B) and red (R) fluorescence can be easily obtained from the Eu-MOFs/perovskites composites by selecting appropriate excitation wavelength.

Interestingly, green (G) fluorescence can also be obtained from the Eu-MOFs/perovskites composites. As shown in Figures 2(c) and 2(d), when exposing the Eu-MOFs/perovskites composites to a UV laser beam of 2 mW under an ambient condition, the blue PL peak under excitation of 365 nm gradually decreases while the green PL peak prominently increases. Finally, the fluorescence color changes from blue to green (Figure 2(e)). The corresponding Commission International de l'Eclairage (CIE) color coordinates of the PL variation are plotted in Figure 2(f), which cover almost the whole color gamut from blue to green. After storage in dark condition for 48 hours, this kind of PL variation is not recoverable. PL quantum yields (QYs) of the B, G, and R emissions are 10.81%, 26.83%, and 64.28%, respectively (Figure S5). Thus, the full color fluorescence is obtained. Meanwhile, during the UV light soaking, the PL spectra excited at 254 nm are also recorded. As shown in Figure S6, the red emission remains almost unchanged. Besides, PL spectra of the Eu-MOFs/perovskites composites are measured under different excitation power intensities. As shown Figure S7, the B, G, and R emissions show linear dependence on excitation power, implying nonlinear effects, such as Auger recombination, saturated absorption, defect filling, and strong electron-phonon coupling are negligible under the relatively low excitation power density.

### 2.3. Mechanism of the Tunable Multicolor Fluorescence

In order to unveil the UV light soaking induced fluorescence variation, the origins of the different emissions are discussed. There is no doubt that the red emission peaks at 579 nm, 591 nm, 618 nm, 648 nm, and 697 nm are consistent with the ^5^D_0_→^7^F_J_ (*J* = 0 − 4) transitions of Eu^3+^ [39]. Besides, it is well accepted that the green emission peak at about 510 to 520 nm is caused by band-to-band transition of CsPbBr_3_. The remaining mystery is the origin of the blue emission. Previous reports have found that blue fluorescence can be generated by Cs_4_PbBr_6_ nanocrystals (Supplementary Note S1). Therefore, we tentatively propose that the blue luminescence comes from radiative defect states of the 0D Cs_4_PbBr_6_. These defects are mainly caused by the substitution of Pb^2+^ by Eu^3+^, as well as the vacancies caused by the reduced halide ligands to satisfy the charge neutrality [40]. Density functional theory (DFT) calculations are performed to calculate the electronic structures of the Eu-doped Cs_4_PbBr_6_ with defects. Trap states locating below the minimum of the conduction band (CBM) are introduced, which make the bandgap down shift to about 2.7 eV (Figure S8, S9). Radiative transitions of these trap states explain the blue emissions [41–44].

**Figure 3:**
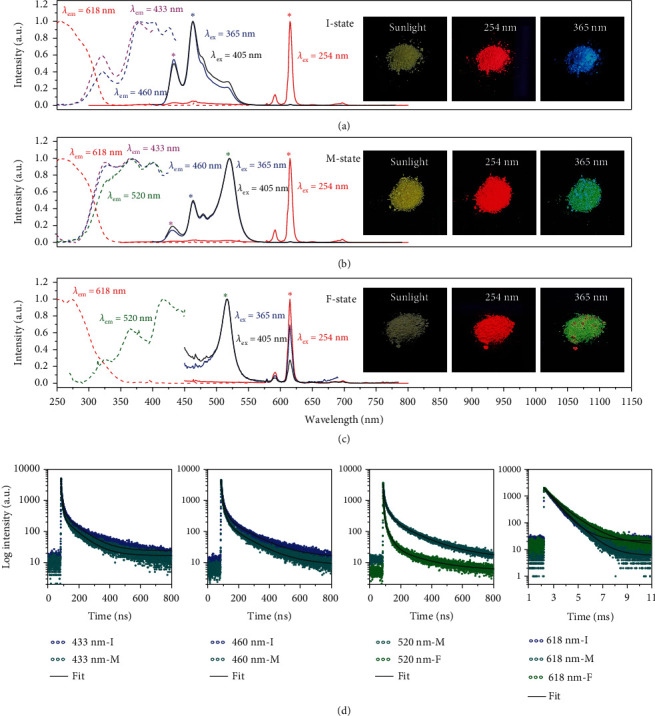
PL, PLE, and time-resolved PL of the composites after different periods of light soaking. (a–c) PLE (dashed line) and PL spectra (solid line) of the Eu-MOFs/perovskites composites at I-state (a), M-state (b), and F-state (c). Inset: corresponding fluorescent photos taken under different excitation lights. (d) PL lifetimes of the emissions at 433 nm in I-state and M-state, at 460 nm in I-state and M-state, at 520 nm in M-state and F-state, and at 618 nm in I-state, M-state, and F-state.

To gain more insight into the origin of the blue emission and the light soaking-induced fluorescence variation, PL of the Eu-MOFs/perovskites composites after different periods of light soaking are investigated in depth. The pristine composite without light soaking is labeled as initial state (I-state), while the samples after a short period of light soaking for 8 minutes and a long period light soaking for 60 minutes are designated as intermediate state (M-state) and final state (F-state), respectively. As shown in insets of Figures 3(a)–3(c), under excitation of 254 nm, all the three states show red fluorescence. The corresponding PL excitation (PLE) spectra monitored at 618 nm contain a broad band emission with a maximum at about 260 nm for all the three states, which is ascribed to the *π*-*π* electron transition of the organic BTC ligands [45]. Combination of the PL and PLE results point out that the f–f transition of Eu^3+^ ion is sensitized by the BTC ligands. When the excitation wavelength is changed to 365 nm, the I-state exhibits blue fluorescence while the other two states exhibit green fluorescence. The PLE spectra corresponding to emissions at 433, 460, and 520 nm are very similar, which contain bands at 320, 370, and 430 nm corresponding to band-to-band transition of the 0D Cs_4_PbBr_6_ and transition of defects. These optical transitions are also discernible in the absorption spectra (Figure S10).

The dynamics for different emissions are analyzed by time resolved PL curves. As shown in Figure 3(d), the PL decay traces of the 433 nm, 460 nm, and 520 nm emissions are fitted by triexponential functions. The fitting details are given in Supplementary Note S2 and Table S1-S4. The average lifetimes of the 433 nm and 460 nm are 15.31 ns and 9.70 ns in the I-state. With increasing light soaking time, these PL lifetimes shorten to 11.19 ns and 8.29 ns, respectively, implying the long-lived defective states are repaired by light soaking. Similarly, from M-state to F-state, PL lifetime of the 520 nm shortens from 24.82 ns to 9.72 ns, respectively, indicating the long-lived traps are removed [46, 47], which is in a good agreement with the steady-state PL spectra. Due to the long energy transfer path from organic ligand to Eu^3+^, the lifetimes of 618 nm emission reach a submicrosecond scale. The PL lifetimes are 0.78 ms to 0.86 ms and 0.99 ms for the I-state, to M-state and F-state, respectively.

According to the spectroscopic analysis above, the light soaking-induced PL variation is correlated to the defect repairing caused by irradiation. The elimination of radiative defects in Cs_4_PbBr_6_ results in quenching of blue emission while the repairing of nonradiative defects in CsPbBr_3_ leads to enhancement of green fluorescence. As a consequence, the blue fluorescence transforms into green fluorescence. The light soaking induced repairing of defects is supported by our XPS measurements (Figure S11 and S12).

The light-driven migration of halide ions is a possible mechanism for defect repairing, which generally completes within tens of seconds with a rapid PL enhancement [48]. However, herein, the fluorescence variation lasts about one hour, which is much slower than the irradiation-induced halide migration. Thus, we propose passivation by irradiation-induced active radicals is responsible for the defect repairing in the perovskites lattice, which lasts tens of minutes to hours [49]. To verify this hypothesis, light soaking of the I-state composites in an oxygen-free environment is conducted. As shown in Figure S13, the PL spectra are almost unchanged during continuous UV irradiation, indicating oxygen plays a critical role in light induced defect repairing. Besides, light irradiation may cause a thermal effect. As shown in Figure S14, under continuous heating at 100°C, the transition from blue to green fluorescence does not occur, excluding the thermal effect. The model of passivation by active radicals is further supported by the DFT calculations. As shown in Figure S15, the formation energy decreases after passivation, indicating these different defects are both the preferred sites for adsorption of active radicals. Moreover, the calculated electronic structure shows that the defect state below the CBM disappears after radical passivation (Figure S16), explaining the quenching of blue fluorescence. Similar enhanced green emission of CsPbBr_3_ by radical passivation has been calculated by Ouyang et al. [50]. Moreover, we would like to emphasize that the phase conversion from 0D to 3D also contributes significantly to the fluorescence variation. We find the photochromic effect is related to moisture (Figure S17, S18). In fact, moisture can induce phase conversion from 0D Cs_4_PbBr_6_ to 3D CsPbBr_3_, which has been verified previously [51, 52]. Our absorption edge highlighted in Figure S10, XRD shown in Figure S19, and HRTEM images shown in Figure S20 confirm the phase conversion from 0D to 3D during the light soaking in the ambient environment. Therefore, moisture-induced phase conversion is also partially responsible for the fluorescence variations observed during light soaking in the ambient environment.

**Figure 4:**
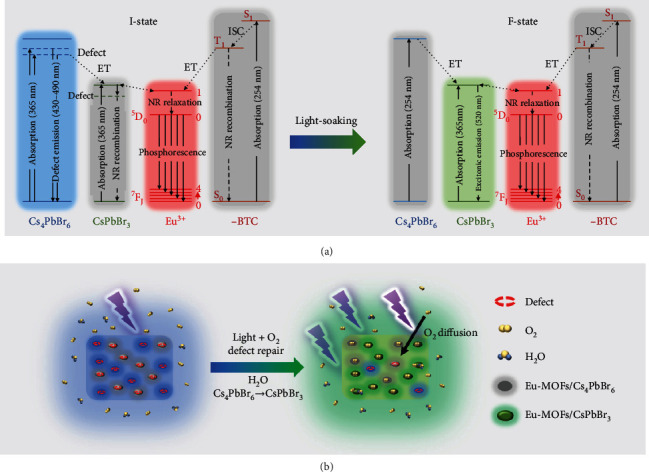
Schemes explaining the PL mechanism and the light soaking-induced fluorescence variations. (a) Schematic diagram explaining the multicolor PL before and after light soaking. S: singlet state; T: triplet state; R: radiative; NR: nonradiative. (b) Schematic diagram of the photochromic process.

According to the above results and discussion, a model to explain the multicolor fluorescence and the tunability is illustrated in Figure 4(a). For the pristine Eu-MOFs/perovskites, under the excitation of ca. 320 to 420 nm, both 0D Cs_4_PbBr_6_ and 3D CsPbBr_3_ components are excited. The 520 nm emission of 3D CsPbBr_3_ is discernible but relatively weak (Figure S21). The fluorescence of the composite is dominated by the blue emission from radiative defects within Cs_4_PbBr_6_, whereas after sufficient light soaking in the ambient environment, both the radiative defects within Cs_4_PbBr_6_ and nonradiative traps within CsPbBr_3_ are passivated. Meanwhile, 0D Cs_4_PbBr_6_ is converted to 3D CsPbBr_3_ due to the interactions with moisture (Figure 4(b)). Consequently, the composites exhibit green emission of CsPbBr_3_. If the excitation wavelength is 240-300 nm, the incident photon is mainly absorbed by the BTC ligands. The excited energy is transferred to the ^5^D_1_ of Eu^3+^ via intersystem crossing (ISC) and then relaxes to the ^5^D_0_ level. The transition from ^5^D_0_ emission level to the ground state generates the red fluorescence [39]. Since the coupling between BTC ligands and perovskites is very weak, direct energy transfer (ET) from BTC to perovskites is not allowed, and blue or green fluorescence cannot be observed in this situation. Thus, under excitation of 254 nm, the composites at different states constantly display red fluorescence.

### 2.4. Applications of the Tunable Multicolor Fluorescence

**Figure 5:**
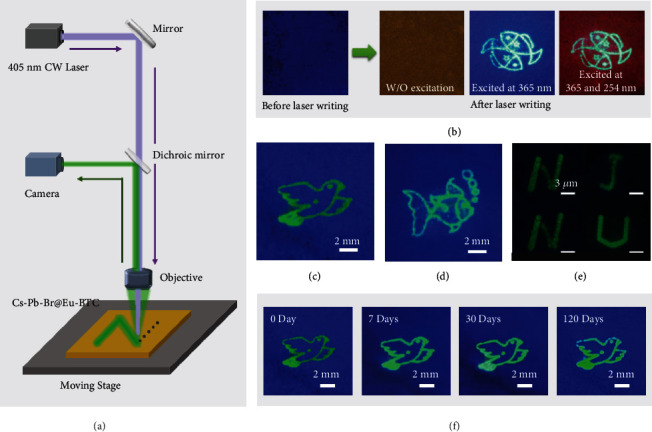
Applications in micropatterning. (a) Schematic of the DLW setup. (b) Photos of the Eu-MOFs/perovskites film before and after DLW. (c, d) Pigeon (c) and fish (d) patterns obtained by the DLW. (e) Fluorescent micrographs of the Eu-MOFs/perovskites film patterned with “N,” “J,” “N,” and “U” characters. (f) Fluorescence patterns during storage in the ambient environment for 120 days (temperature of 10-40°C and humidity of 30%-70%).

The unique light soaking-induced permanent fluorescence variation builds the foundation for laser fabrication. The Eu-MOFs/perovskites powders are pressed into a flat film. A direct laser writing (DLW) setup is used to pattern on the fluorescent film (Figure 5(a)) [16]. A 405 nm laser beam of 2 mW is focused on the film via an objective. As shown in Figure 5(b), before laser writing, the whole film exhibits blue fluorescence. After DLW, the processed region turns into bright green fluorescence; thus, a pattern is obtained. The pattern cannot be observed without an UV excitation beam, which creates a unique security mechanism and is very significant for anticounterfeiting and steganography. Moreover, when the film is jointly excited by 365 and 254 nm lights, the background color changes from blue to red due to the exceptional excitation-dependent PL, which further increases the difficulty of imitation, thus improving the security for anticounterfeiting. Figures 5(c) and 5(d) demonstrate that different fluorescence patterns can be easily drawn. In addition, characters at a microscale can be conveniently written by the DLW method (Figure 5(e)). Figure 5(f) shows that the polymer-protected fluorescence patterns are still readable after storage in the ambient environment for 120 days.

**Figure 6:**
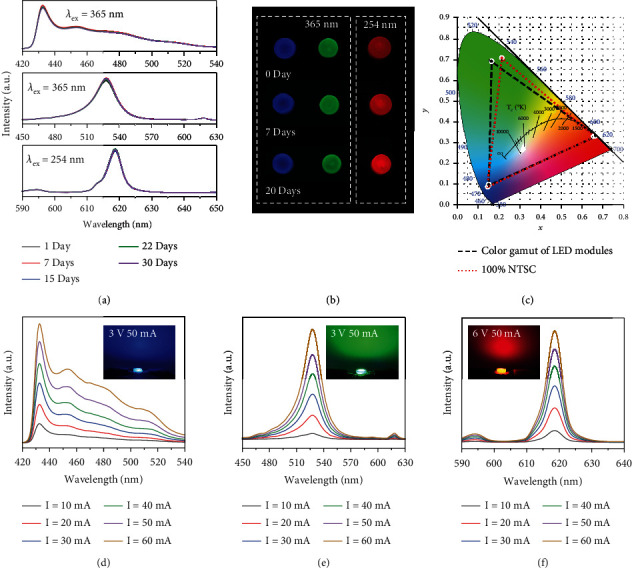
Applications in LED modules. (a) PL spectra of the Eu-MOFs/perovskites encapsulated by PMMA after long-term storage (temperature of 10-40°C and humidity of 30%-70%). (b) Fluorescent photos of the Eu-MOFs/perovskites encapsulated by PMMA after storage for 0, 7, and 20 days, excited at 365 nm and 254 nm. (c) Color gamut of the three LED modules. (d–f) EL spectra of the trichromatic LED modules. Insets are photos of the lit-up LEDs.

Moreover, the Eu-MOFs/perovskites composites can realize trichromatic emissions, showing enormous application foreground at the aspects of LEDs and display. To verify the feasibility, the I-state and F-state Eu-MOFs/perovskites composites are encapsulated by polymethyl methacrylate (PMMA) to yield LED color converters, which are then combined with 365 nm and 265 nm InGaN chips. As shown in Figures 6(a) and 6(b), light soaking-induced variation is prevented due to the isolation of the Eu-MOFs/perovskites from the air. After storage in the ambient environment for 30 days, the PL intensities of the three LED modules almost keep constant without spectral shift, indicating the outstanding long-term stability with the encapsulation. The CIE coordinates of the LED modules are shown in Figure 6(c), which cover 95.9% of the color gamut introduced in 1951 by the National Television System Committee (NTSC) [53]. Electroluminescence (EL) spectra of the trichromatic LED modules are shown in Figures 6(d)–6(f). The UV emission of the InGaN chip is successfully converted to RGB emissions. With the increase of the driving current, the EL intensities of LED modules linearly increase, without changing the peak positions and band widths. When the current is 50 mA, the brightness of the blue, green, and red LED modules reaches 621.0 cd/m^2^, 705.8 cd/m^2^, and 1253.2 cd/m^2^, respectively. More parameters related to the LED modules are shown in Table S5.

## 3. Conclusion

In summary, a strategy is innovated for construction of tunable multicolor luminescence by manipulating the radiative defects and structural dimensionality. The fluorescent composite containing Eu-BTC MOFs, 0D Cs_4_PbBr_6_, and 3D CsPbBr_3_ is synthesized by a simple solvent method. The as-prepared Eu-MOFs/perovskites composites emit blue and red fluorescence when excited at 365 nm and 254 nm, respectively. Under continuous light soaking, the blue fluorescence gradually quenches while the green fluorescence rapidly rises. Thus, the RGB emissions are obtained. The blue emission is attributed to radiative defects within Cs_4_PbBr_6_. The green emission originates from band-to-band transition of CsPbBr_3_. And ^5^D_0_→^7^F_J_ transitions of Eu^3+^ is responsible for the red emission. After light soaking in the ambient environment, both the radiative defects within Cs_4_PbBr_6_ and nonradiative traps within CsPbBr_3_ are passivated. Meanwhile, Cs_4_PbBr_6_ is converted to CsPbBr_3_ due to the interactions with moisture. Consequently, the blue fluorescence turns into green. Based on the photochromic process, microfluorescence patterns are depicted by DLW, paving the way for applications in anticounterfeiting and microsteganography. Moreover, trichromatic LED modules are fabricated by using the Eu-MOFs/perovskites composites as color-converting layers, which almost cover the entire color gamut demanded by NTSC. Fluorescence of the color-converting layers with the encapsulation are very stable during working and long-term storage, exhibiting great application potential in lighting and display.

## 4. Methods

### 4.1. Materials

Lead bromide (PbBr_2_, 99%, Aladdin), hexanoic acid (99.9%, Aladdin), octylamine (99.9%, Aladdin), N,N-dimethylformamide (DMF, 99%, Aladdin), cesium carbonate (Cs_2_CO_3_, 99%, Aladdin), octadecene (ODE 99.9%, Aladdin), oleic acid (OA 90%, Aldrich), oleylamine (OAm, 90%, McLean), Eu(NO_3_)_3_·6H_2_O (99.9%, Aladdin), 1,3,5-trimesic acid (H_3_BTC, 98%, Aladdin), sodium acetate (NaAc, 99%, Aladdin), toluene (99%, Aladdin), and ethyl acetate (99.9%, Aladdin) are used. All reagents were used without any further purification.

### 4.2. Preparation of Eu-MOFs/Perovskites Composites

Cs_2_CO_3_ powders (0.407 g, 1.25 mmol) were loaded into a 100 mL 3-neck flask with ODE (18 mL) and OA (1.74 mL), dried for 1 h at 120°C, and then heated to 150°C under N_2_ atmosphere until all Cs_2_CO_3_ powders were reacted. PbBr_2_ (0.183 g, 0.5 mmol) was dissolved in a solution containing dimethylformamide (10 mL), caproic acid (0.58 g, 5 mmol), and octylamine (0.645 g, 5 mmol). After Cs-oleate (0.5 mL) was added to the mixture, Eu(NO_3_)_3_·6H_2_O (0.089 g 0.2 mmol) and NaAc (0.6 mL) were added to the mixture to form a metal precursor solution. Then, tricarboxylic acid (H_3_BTC) (0.1 g, 0.50 mmol) was added to the metal precursors containing Eu^3+^ ions. After continuously stirring for 12 hours at room temperature, 1 mL above mixture was added to 10 mL toluene, and the Eu-MOFs/perovskites composite products were collected by centrifugation, washed by ethyl acetate for several times, and dried naturally for 12 hours.

### 4.3. Preparation of Eu-MOFs/Perovskites Composites@PMMA

In the fume hood, 1 g PMMA solid powders were added to a 40 mL flask containing 10 mL toluene solution. Then, mixture was stirred vigorously at 40°C. After the PMMA was completely dissolved, different states of Eu-MOFs/perovskites composites were added to the mixed solution by continuous stirring. Then, the resulting product was poured into the prepared mold and dried naturally for 24 h in a ventilated area.

### 4.4. Preparation of LED Modules

The Eu-MOFs/perovskites composites@PMMA with different colors of fluorescence were directly coupled to the InGaN blue light-emitting chip to form the LED devices. The emission wavelengths of the InGaN chip were 365 nm and 265 nm. In addition, to avoid the leakage of UV light, the edges of the device were filled with opaque silica gel.

### 4.5. Characterizations

XRD patterns were obtained by D/max 2500/PC rotating target X-ray diffractometer. XPS data were acquired from ESCALAB Xi+ X-ray photoelectron spectrometer. SEM images were recorded by Apreo 2S electron microscope. TEM images were recorded by JEM-2100F electron microscope (JOEL). The compositional distribution was investigated by EDS equipped on the TEM. The absorption spectra were obtained by using Shimazu UV2600 UV-vis spectrophotometer. PL spectra were obtained by a Maya 2000 Pro high-sensitivity spectrometer (Ocean Optics). The PL decay curves, PLE spectra, and PLQYs were measured on Horiba Jobin Yvon FM-4P-TCSPC fluorescence spectrophotometer.

### 4.6. DFT Calculations

The theoretical calculation was conducted by using the DFT within the generalized gradient approximation under CASTEP package. A kinetic energy cutoff of 489 eV was used to represent the single-particle wave functions. The geometric optimization was carried out with convergence tolerances of 2 × 10^−5^ eV for energy, 0.05 eV/Å for maximum force, and 0.002 Å for maximum displacement.

## Data Availability

Data supporting the findings of this study are available in the main text or the supplementary information. Additional data related to this paper may be requested from the authors.

## Supplementary Materials

Supplementary Materials Supplementary Note S1: proof materials used to confirm the origin of the blue emission. Supplementary Note S2: numerical fittings on the time resolved PL decay traces. Table S1: fitting parameters of the decay curves for the 433 nm emission. Table S2: fitting parameters of the decay curves for the 460 nm emission. Table S3: fitting parameters of the decay curves for the 520 nm emission. Table S4: fitting parameters of the decay curves for the 618 nm emission. Table S5: performance parameters of the LED modules. Figure S1: SEM image of the Eu-MOFs/perovskites composites. Figure S2: EDS spectrum of the Eu-MOFs/perovskites composites. Figure S3: TEM (a) and HRTEM (b–d) images of the Eu-MOFs/perovskites composites. Figure S4: XPS spectra of the Eu-MOFs/perovskites composites. Figure S5: PL spectra with excitation references. Figure S6: light soaking experiment of Eu-MOFs/perovskites composites excited by 254 nm. Figure S7: the optical properties of the Eu-MOFs/perovskites composites under different excitation intensities. Figure S8: (a) the band structure of the pristine Cs_4_PbBr_6_; (b) the calculated PDOS of pristine Cs_4_PbBr_6_. Figure S9: structural diagram and PDOS of Cs_4_PbBr_6_ with Eu+V_Br_ and Eu+I_Br_. Figure S10: absorption spectra of the Eu-MOFs/perovskites composites. Figure S11: XPS spectra of the Eu-MOFs/perovskites composites. Figure S12: XPS spectra of Pb 4f (a), Br 3d (b) and Cs 3d (c) in Eu-MOFs/perovskites composites. Figure S13: light soaking experiment of Eu-MOFs/perovskites composites in a vacuum environment. Figure S14: heating experiment of Eu-MOFs/perovskites composites. Figure S15: formation energies of defects before and after adsorption of O_2_. Figure S16: structural diagram and PDOS of Cs_4_PbBr_6_ with Eu+V_Br_ and Eu+I_Br_ after O_2_ adsorption. Figure S17: light soaking experiment of MOFs/perovskites composites in a dry environment. Figure S18: spray treatment experiment of Eu-MOFs/perovskites composites. Figure S19: XRD patterns of Eu-MOFs/perovskites composites in different states. Figure S20: HRTEM images of the Eu-MOFs/perovskites composites in I-state (a-c) and F-state (d, e). Figure S21: Gaussian fitting of the PL spectrum of the pristine Eu-MOFs/perovskites composites.
