# Supplementary material for: Tunable Multicolor Fluorescence of Perovskite-Based Composites for Optical Steganography and Light-Emitting Devices
Source: Research (Wash D C). 2022 Sep 13;2022:9896548. doi: 10.34133/2022/9896548 (PMC9513829; doi:10.34133/2022/9896548)
Supplement: Supplementary Materials — Supplementary Note S1: proof materials used to confirm the origin of the blue emission. Supplementary Note S2: numerical fittings on the time resolved PL decay traces. Table S1: fitting parameters of the decay curves for the 433 nm emission. Table S2: fitting parameters of the decay curves for the 460 nm emission. Table S3: fitting parameters of the decay curves for the 520 nm emission. Table S4: fitting parameters of the decay curves for the 618 nm emission. Table S5: performance parameters of the LED modules. Figure S1: SEM image of the Eu-MOFs/perovskites composites. Figure S2: EDS spectrum of the Eu-MOFs/perovskites composites. Figure S3: TEM (a) and HRTEM (b–d) images of the Eu-MOFs/perovskites composites. Figure S4: XPS spectra of the Eu-MOFs/perovskites composites. Figure S5: PL spectra with excitation references. Figure S6: light soaking experiment of Eu-MOFs/perovskites composites excited by 254 nm. Figure S7: the optical properties of the Eu-MOFs/perovskites composites under different excitation intensities. Figure S8: (a) the band structure of the pristine Cs4PbBr6; (b) the calculated PDOS of pristine Cs4PbBr6. Figure S9: structural diagram and PDOS of Cs4PbBr6 with Eu+VBr and Eu+IBr. Figure S10: absorption spectra of the Eu-MOFs/perovskites composites. Figure S11: XPS spectra of the Eu-MOFs/perovskites composites. Figure S12: XPS spectra of Pb 4f (a), Br 3d (b) and Cs 3d (c) in Eu-MOFs/perovskites composites. Figure S13: light soaking experiment of Eu-MOFs/perovskites composites in a vacuum environment. Figure S14: heating experiment of Eu-MOFs/perovskites composites. Figure S15: formation energies of defects before and after adsorption of O2. Figure S16: structural diagram and PDOS of Cs4PbBr6 with Eu+VBr and Eu+IBr after O2 adsorption. Figure S17: light soaking experiment of MOFs/perovskites composites in a dry environment. Figure S18: spray treatment experiment of Eu-MOFs/perovskites composites. Figure S19: XRD patterns of Eu-MOFs/perovskites composit [file 9896548.f1.docx]

**Tunable multicolor fluorescence of** **perovskite-based composites for optical steganography and light-emitting devices**

*Kewei Ma,^1^* *Qingfeng Gui,^2^ Cihui Liu,^1^ Yunyi Yang,^3^ Fangjian Xing,^1^ Yunsong Di,^1^ Xiaoming Wen,^3^ Baohua Jia,^3,4^ Zhixing Gan^1,5*^*

1. Center for Future Optoelectronic Functional Materials, School of Computer and Electronic Information/School of Artificial Intelligence, Nanjing Normal University, Nanjing 210023, P. R. China.

2. College of Naval Architecture and Ocean Engineering, Jiangsu Maritime Institute, Nanjing 211170, P. R. China.

3. Centre for Translational Atomaterials, School of Science, Swinburne University of Technology, John Street Hawthorn, VIC 3122, Australia.

4. School of Science, RMIT University, Melbourne, 3000, VIC, Australia.

5. College of Materials Science and Engineering, Qingdao University of Science and Technology, Qingdao 266042, P. R. China.

**Keywords:** perovskites, photoluminescence, light soaking, direct laser writing, anti-counterfeiting

**
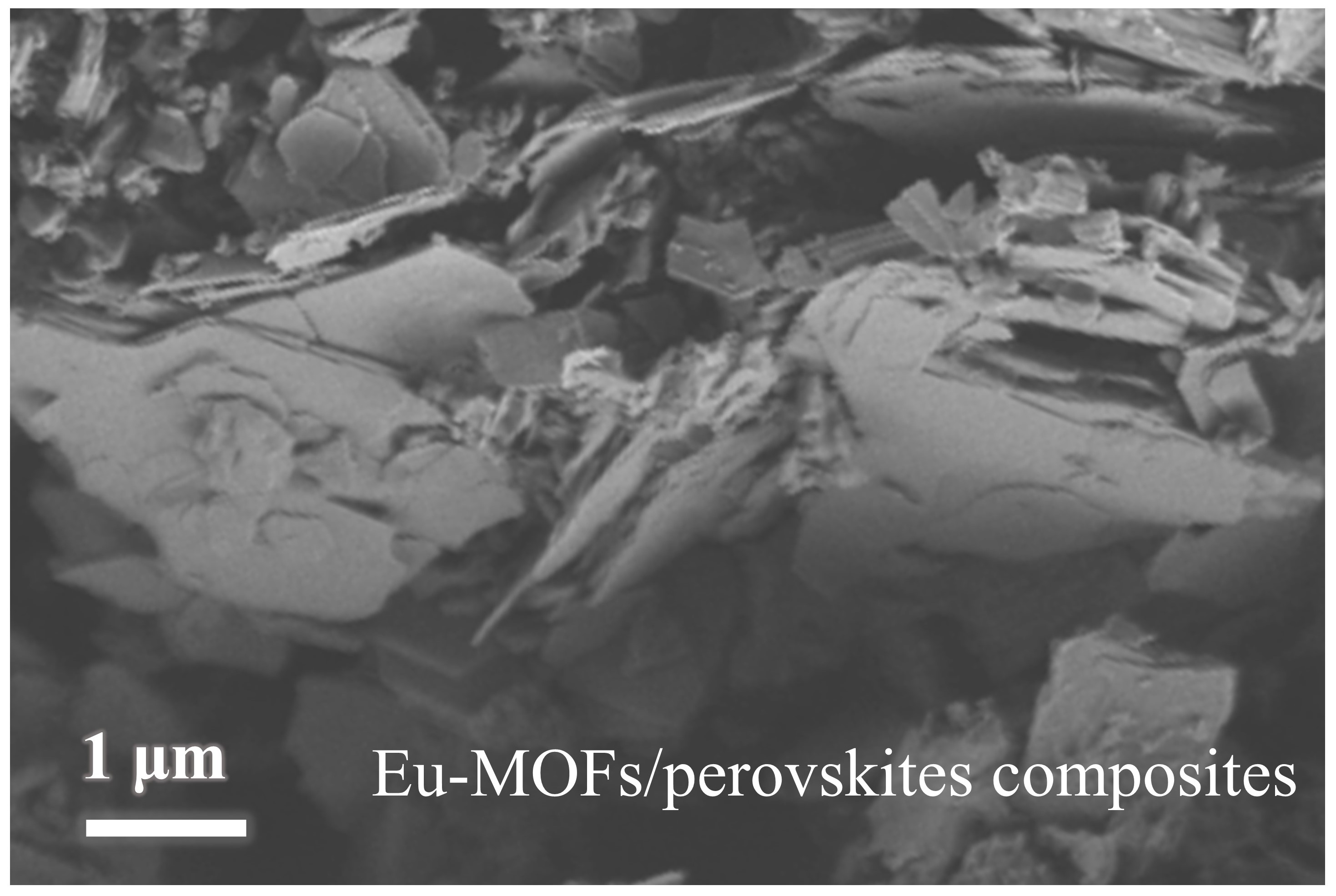
**

**Figure S1** SEM image of the Eu-MOFs/perovskites composites.


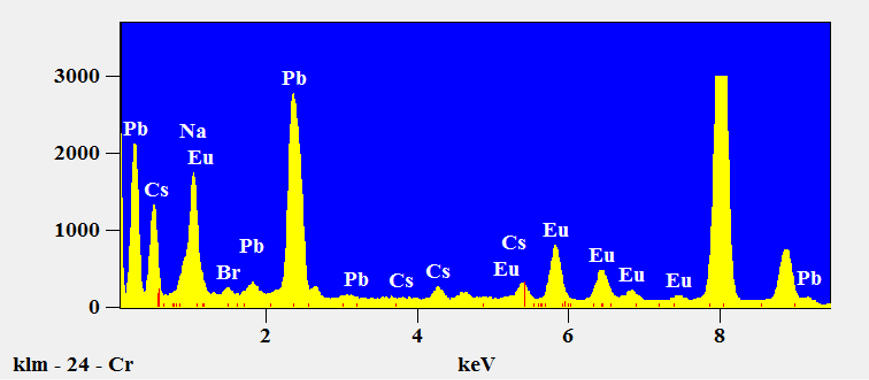


**Figure S2** EDS spectrum of the Eu-MOFs/perovskites composites.


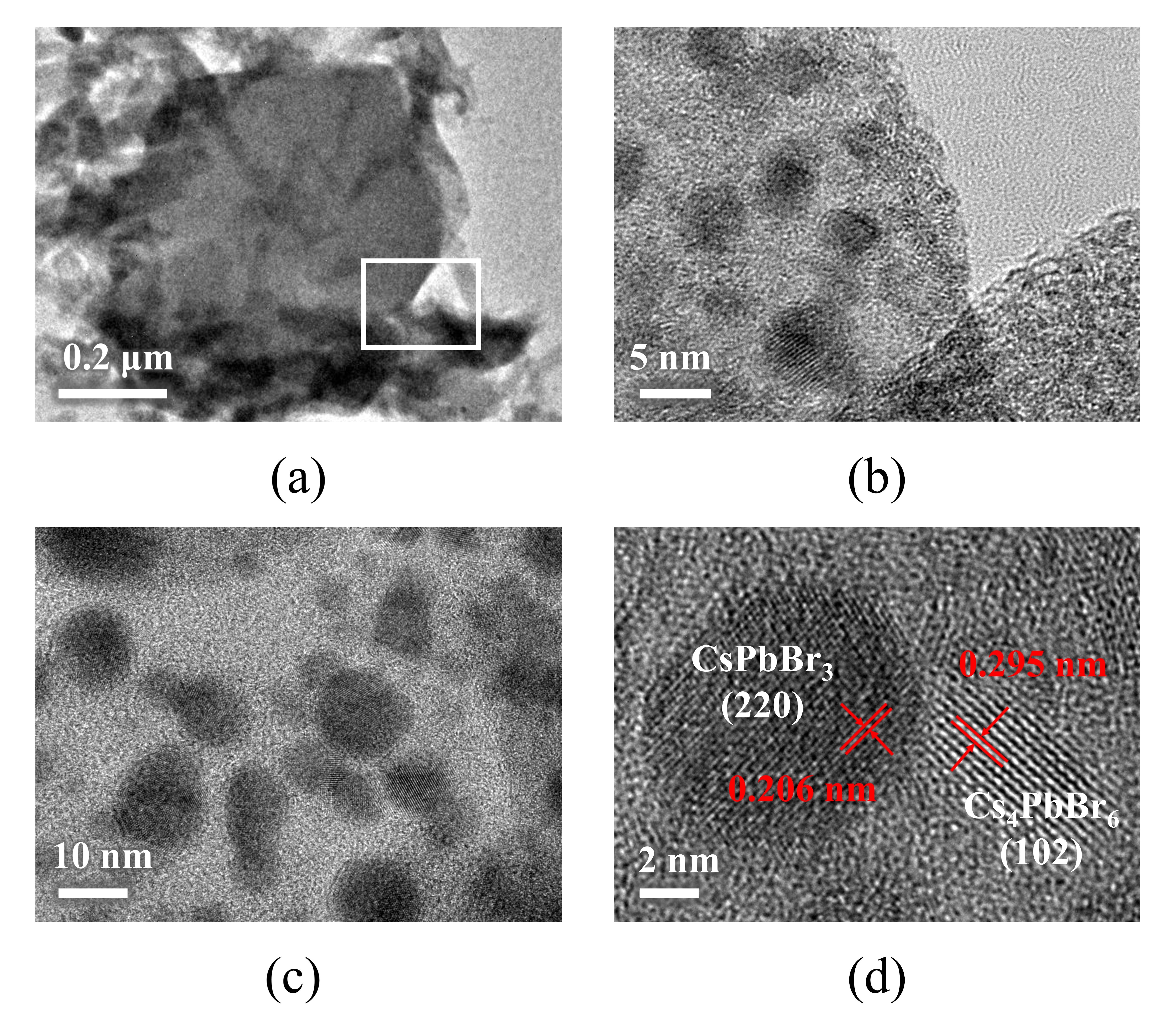


**Figure S3** (a) TEM image of the Eu-MOFs/perovskites composites. (b) HRTEM image of the Eu-MOFs/perovskites composites in the selected area shown in panel (a). (c, d) HRTEM image of the Eu-MOFs/perovskites composites.





**Figure S4** XPS spectra of the Eu-MOFs/perovskites composites. (a-e) C 1s (a),Cs 3d (b), Pb 4f (c), Br 3d (d), Eu 3d (e). (f) Full survey spectrum.


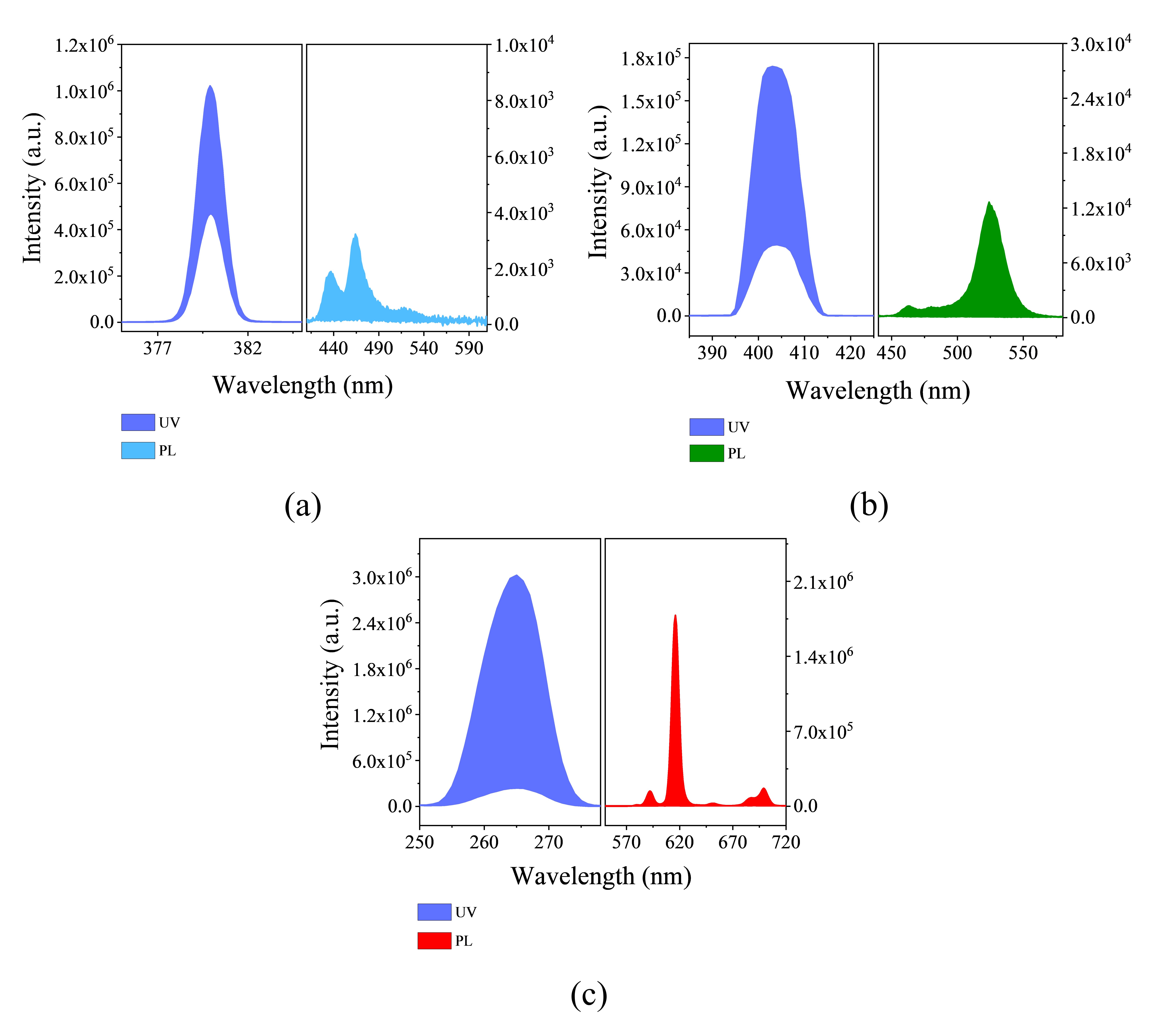


**Figure S5** (a-c) PL spectra with UV excitation references used to calculate QYs of B (a), G (b), R (c) emissions.


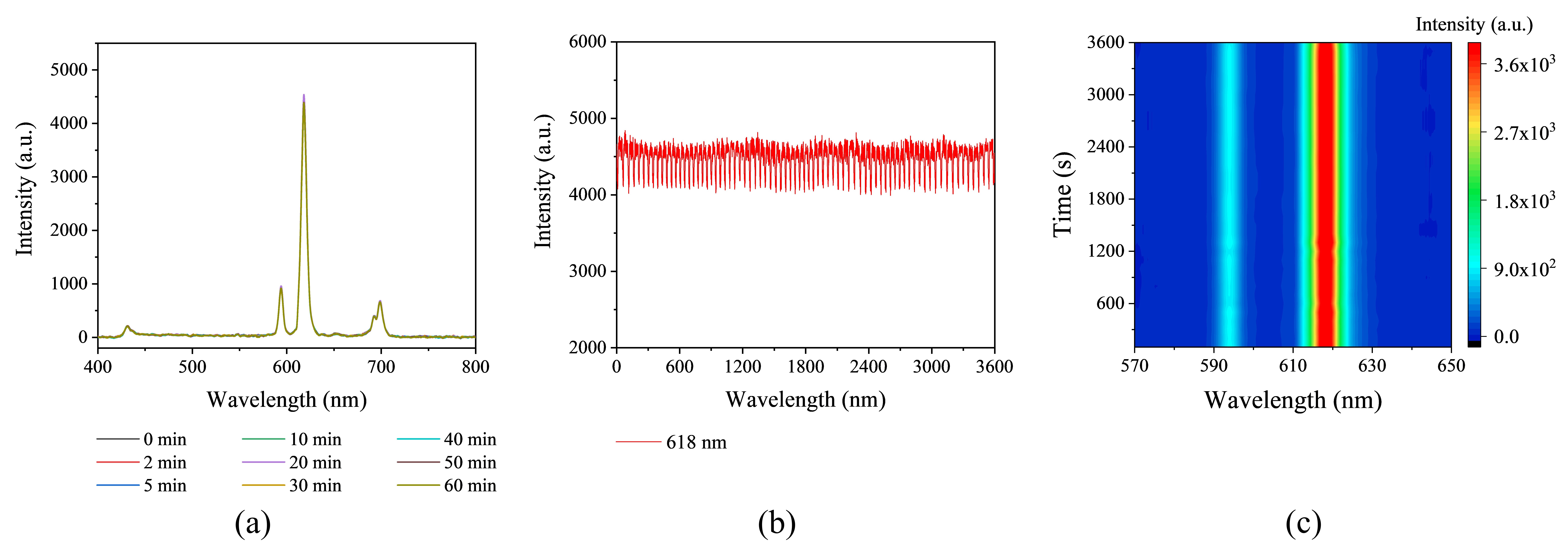


**Figure S6** (a) PL spectra of the Eu-MOFs/perovskites composites excited at 254 nm during light-soaking. (b) Intensity of the 618 nm-PL band varies with time. (c) Pseudocolor PL contour mapping excited at 254 nm during light-soaking.


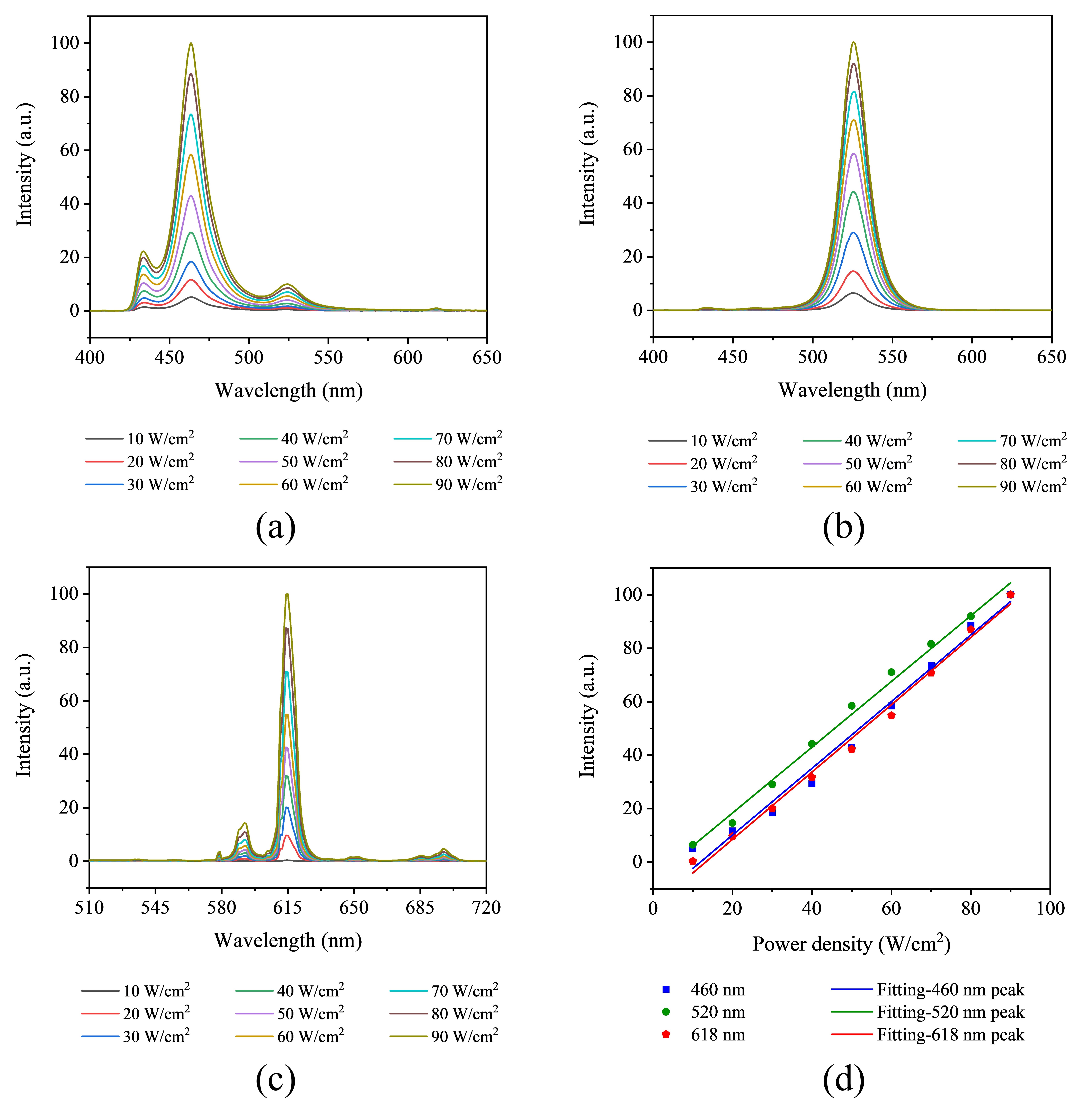


**Figure S7** (a-c) Blue (a), green (b) and red (c) PL spectra of the Eu-MOFs/ perovskite composites excited at different power densities. (d) Dependence of PL intensity on excitation power density.

**Supplementary Note S1.**

As reported by Chen et al. [1], the growth process of Cs_4_PbBr_6_ crystals is divided into three stages (stage I, 0–3 h; stage II, 3–12 h; stage III, 12–24 h). During the first and second stages, the Cs_4_PbBr_6_crystals show the blue emissions at about 464 nm and 490 nm. Likewise, Wang et al designed an in-situ detection system to probe the PL spectral evolution of Cs_4_PbBr_6_ during the nucleation growth [2]. Two distinct stages are observed. In the first stage, Cs_4_PbBr_6_ mainly emits at about 490 nm. Besides, Sun et al. [3] synthesized Cs_4_PbBr_6_ crystals with blue emission at 467 nm in slow-cooling crystallization process. During the aging process, the blue fluorescence gradually changes into green. Cs_4_PbBr_6_ perovskite nanocrystals with emissions at 405 nm also have been by synthesized a supersaturated recrystallization process [4]. According to the previous reports [1-3], the Cs_4_PbBr_6_ perovskite can indeed emit at 405 to 490 nm. And the blue emission is generally instable, which is in good agreement with our observation. Combining with our spectral analysis, control experiments, structural characterizations, and theoretical calculations, it is very reasonable to propose that the blue luminescence comes from the 0D Cs_4_PbBr_6_.


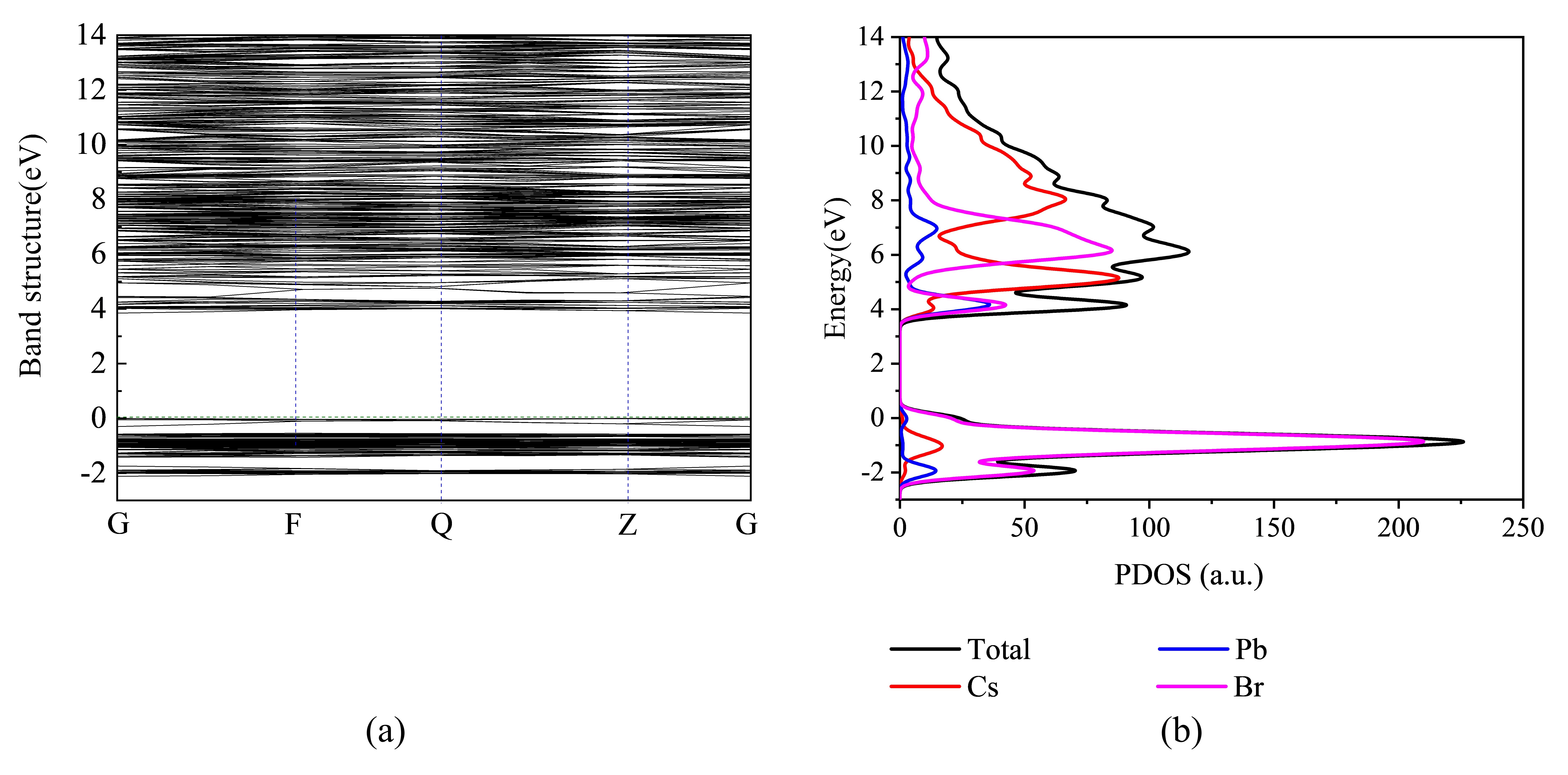


**Figure S8** (a) Band structure of the pristine Cs_4_PbBr_6_. (b) Calculated partial densities of states (PDOS) of the pristine Cs_4_PbBr_6_.

**
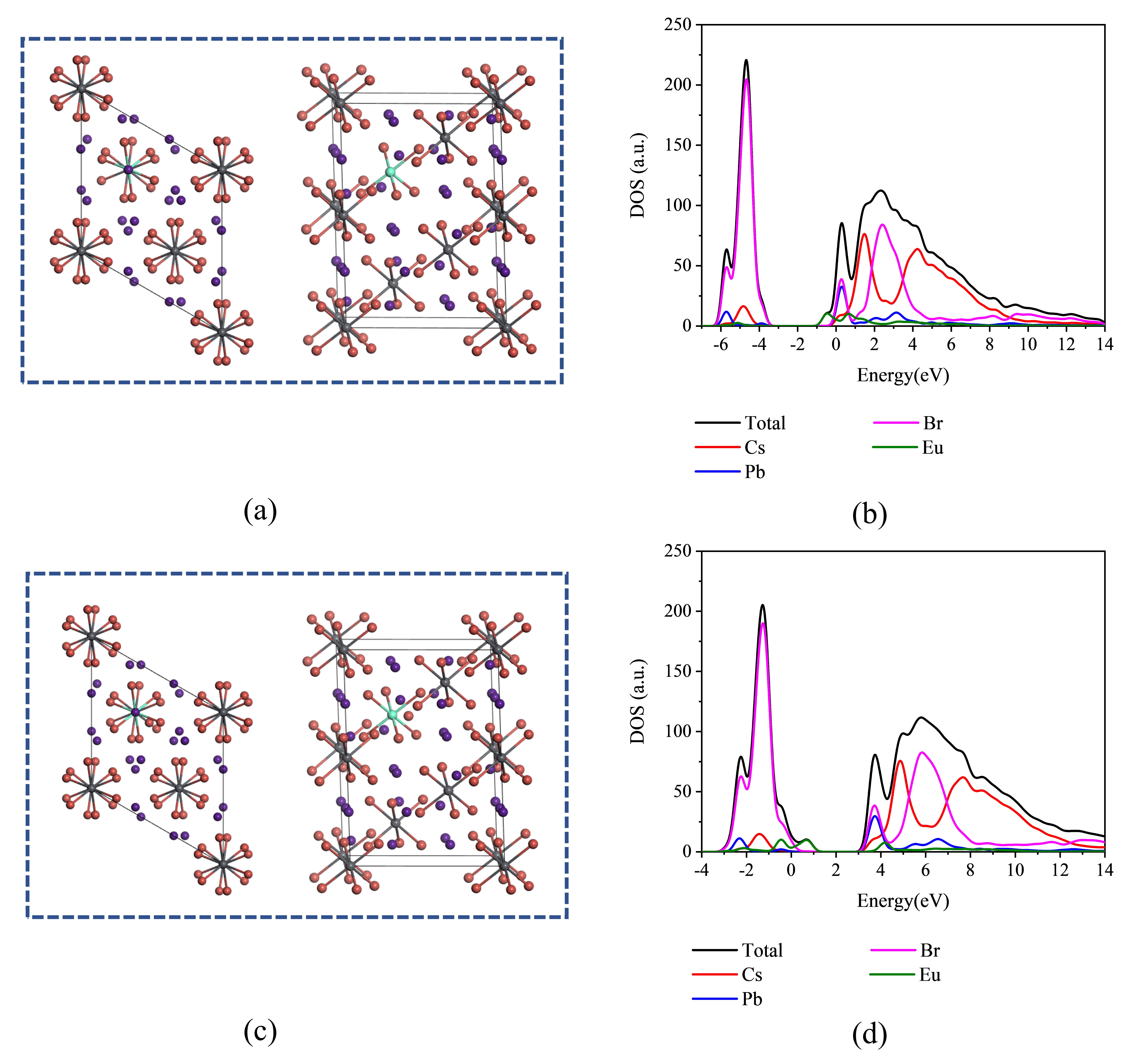
**

**Figure S9** (a) Structural diagram of Cs_4_PbBr_6_ with Eu+V_Br_. (b) Calculated PDOS of the Cs_4_PbBr_6_ with Eu+V_Br_. (c) Structural diagram of the Cs_4_PbBr_6_ with Eu+I_Br_. (d) Calculated PDOS of the Cs_4_PbBr_6_ with Eu+I_Br_.

The calculated bandgap of the pristine Cs_4_PbBr_6_ is about 3.85 eV (Figure S8), which matches well with the experimental value [5-7]. We mainly considered two different configurations, i.e. doping Eu accompanying with a Br vacancy (Eu+V_Br_) and doping Eu accompanying with an interstitial Br (Eu+I_Br_), which are the defects frequently appear in different perovskites [8-11]. As shown in Figure S9, a trap state locating below the minimum of the conduction band (CBM) is introduced in the configuration of Eu+V_Br_, which makes the bandgap down shift to about 2.7 eV. The transition of this trap state can explain the blue emission. Similarly, for the configuration of Eu+I_Br_, bandgap is also narrowed. Therefore, the blue fluorescence ranging from 430 to 460 nm originates from the different defect configurations of Cs_4_PbBr_6_ [8, 12-14].


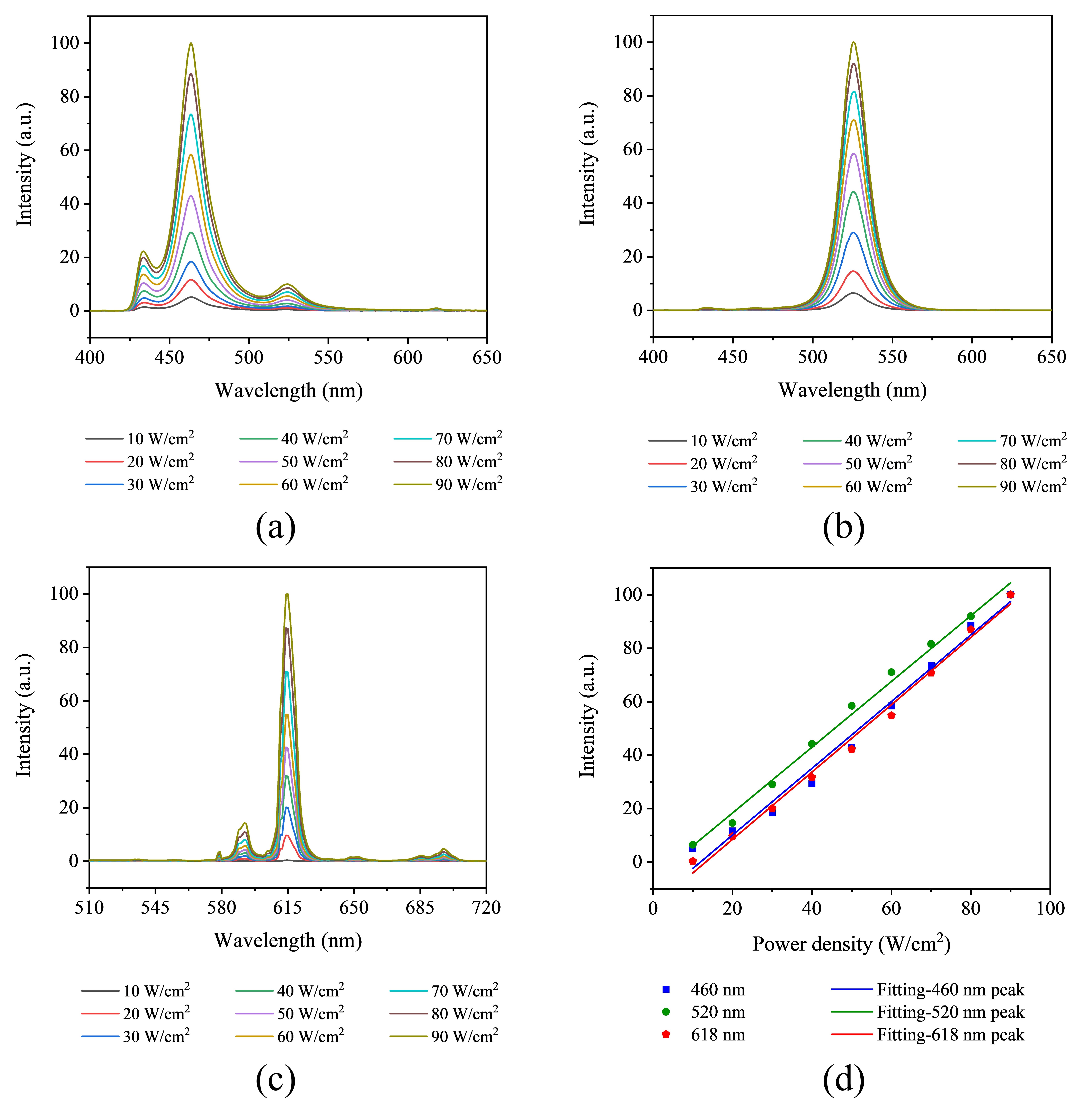


**Figure S10** (a) Absorption spectra of the Eu-MOFs/perovskites composites under different states. (b) Highlights of the absorption band edges in the wavelength range of 430 - 550 nm.

From the ultraviolet-visible (UV-vis) absorption spectra (Figure S10), the absorption characteristics of 320 nm and 520 nm belonging to the Cs_4_PbBr_6_ and CsPbBr_3_ are evident [15, 16], further indicating the coexistence of different dimensional components of perovskites. In addition, absorption at 433 and 460 nm caused by defect states are also observed. With the increase of light-soaking, the absorbance at wavelengths of 350-500 nm gradually becomes smooth due to the decreasing of absorption at 430 and 460 nm, while the edges at 520 nm becomes more prominent.

**Supplementary Note S2. Numerical fittings on the time resolved PL decay traces.**

Time-resolved PL decay curves of the 433 nm, 460 nm, and 520 nm emissions were fitted to a triexponential function [17],

$f\left( t \right)=B_{1}\exp\left( -\frac{t}{\tau_{1}} \right)+B_{2}\exp\left( -\frac{t}{\tau_{2}} \right)+B_{3}\exp\left( -\frac{t}{\tau_{3}} \right)$ (1)

The average lifetimes were calculated using

$\tau_{avg}=(B_{1}\tau_{1}+B_{2}\tau_{2}+B_{3}\tau_{3})/(B_{1}+B_{2}+B_{3})$  (2)

Time-resolved PL decay curves of the 618 nm emission were fitted to a bi-exponential function,

$f\left( t \right)=B_{1}\exp\left( -\frac{t}{\tau_{1}} \right)+B_{2}\exp\left( -\frac{t}{\tau_{2}} \right)$ (3)

The average lifetimes were calculated using

$\tau_{avg}=(B_{1}\tau_{1}+B_{2}\tau_{2})/(B_{1}+B_{2})$ (4)

The blue and green emissions contain three lifetimes, i.e. $\tau_{1}$ of ca. 1-5 ns, $\tau_{2}$ about of 10-20 ns, and $\tau_{3}$ about 150 ns. The short $\tau_{1}$ is attributed to the fast nonradiative trapping, which is usually faster than excitonic recombination [18-20]. $\tau_{2}$ is ascribed to the radiative transition [18, 19]. The long $\tau_{3}$ is unusual, which is possibly caused by recombination of delocalized carriers originally trapped by the long-lived traps[19-22]. Many different groups have revealed the existence of long-lived traps in lead halide perovskites. For example, Chirvony et al [20]. constructed a delayed luminescence model to explain the long-lived components. They proposed that the role of the traps is dual. Besides the well accepted non-radiative recombination with very short lifetime, traps also act as a carrier reservoir. And the long-lived lifetime is determined by the depth of the trap states, lying from a few tens to hundreds meV below the emitting excitonic state. The traps and exciton states are coupled through the capture-de-trapping process, resulting in the accumulation of electrons in the traps with a long lifetime (the average long-lived lifetime is about 150 ns). Similar conclusions have been proposed in other reports [21, 22].

The average lifetimes of the 433 nm and 460 nm are 15.31 ns and 9.70 ns in the I-state. With increasing of light-soaking time, these PL lifetimes shortens to 11.19 ns and 8.29 ns, respectively, due to the decreased amplitude of $\tau_{3}$, implying the long-lived defective states are repaired by light-soaking. The lifetime of 24.82 ns is abnormally longer than the lifetime of excitonic recombination, which is caused by the long-lived traps. After light soaking, the lifetime of 9.72 ns is close to the normal lifetime of CsPbBr_3_ widely reported previously [23, 24], indicating the long-lived traps are removed, which is in good agreement with the steady-state PL spectra.

**Table S1** Fitting parameters of the decay curves for the 433 nm emission.

|  | **τ_1_ (ns)** | **τ_2_ (ns)** | **τ_3_ (ns)** | **B_1_** | **B_2_** | **B_3_** | **τ_avg_ (ns)** |
| --- | --- | --- | --- | --- | --- | --- | --- |
| **433 nm-I** | 1.19 | 10.33 | 170.32 | 3069.88 | 1369.88 | 323.53 | **15.31** |
| **433 nm-M** | 1.19 | 10.33 | 172.69 | 2570.26 | 1593.63 | 167.56 | **11.19** |

**Table S2** Fitting parameters of the decay curves for the 460 nm emission.

|  | **τ_1_ (ns)** | **τ_2_ (ns)** | **τ_3_ (ns)** | **B_1_** | **B_2_** | **B_3_** | **τ_avg_ (ns)** |
| --- | --- | --- | --- | --- | --- | --- | --- |
| **460 nm-I** | 4.56 | 21.49 | 148.22 | 17615.65 | 1560.23 | 521.13 | **9.70** |
| **460 nm-M** | 4.44 | 21.35 | 157.05 | 16774.28 | 1670.55 | 287.85 | **8.29** |

**Table S3** Fitting parameters of the decay curves for the 520 nm emission.

|  | **τ_1_ (ns)** | **τ_2_ (ns)** | **τ_3_ (ns)** | **B_1_** | **B_2_** | **B_3_** | **τ_avg_ (ns)** |
| --- | --- | --- | --- | --- | --- | --- | --- |
| **520 nm-M** | 1.35 | 14.08 | 153.77 | 4572.53 | 2772.55 | 1063.36 | **24.82** |
| **520 nm-F** | 1.22 | 13.12 | 154.89 | 3988.49 | 3171.73 | 159.30 | **9.72** |

**Table S4** Fitting parameters of the decay curves for the 618 nm emission.

|  | **τ_1_ (ms)** | **τ_2_ (ms)** | **B_1_** | **B_2_** | **τ_avg_ (ms)** |
| --- | --- | --- | --- | --- | --- |
| **618 nm-I** | 0.69 | 1.55 | 1.76×10^-03^ | 1.96×10^-02^ | **0.78** |
| **618 nm-M** | 0.78 | 1.64 | 1.75×10^-03^ | 1.85×10^-02^ | **0.86** |
| **618 nm-F** | 0.68 | 1.4 | 1.10×10^-03^ | 8.50×10^-02^ | **0.99** |


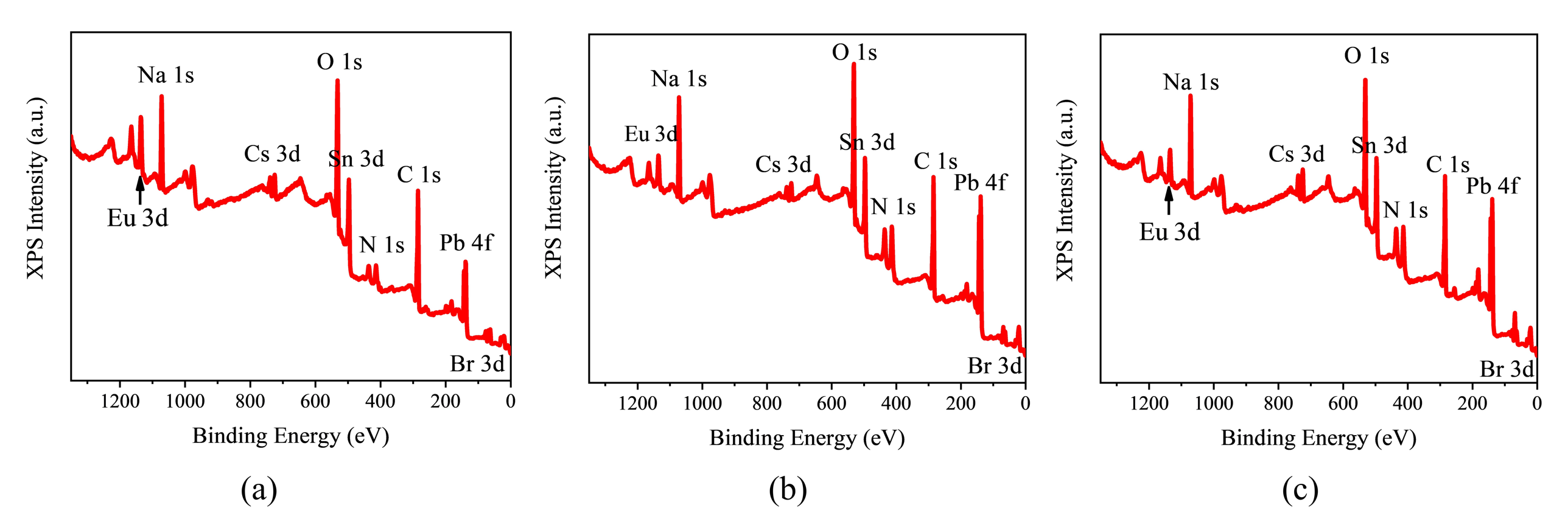


**Figure S11** XPS spectra of the Eu-MOFs/perovskites composites at I-state (a), M-state (b) and F-state (c).


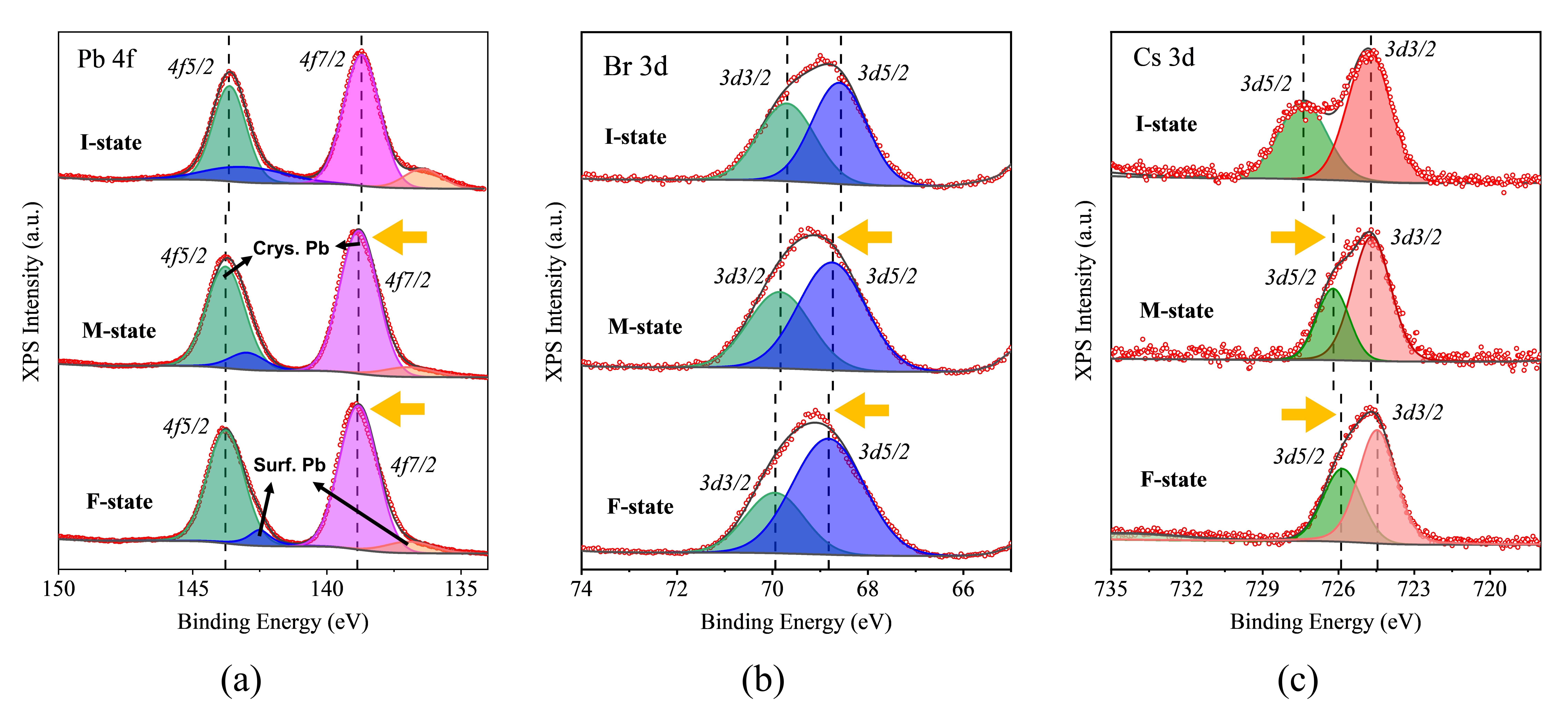


**Figure S12** XPS spectra of the Eu-MOFs/perovskites composites at different states. Pb 4f (a), Br 3d (b) and Cs 3d (c).

As shown in Figure S12, the Pb 4f XPS core-level spectra contain two dominant peaks of Pb 4f_5/2_ (143.6 eV) and Pb 4f_7/2_ (138.7 eV), accompanying with two side peaks at 142.9 and 136.5 eV. The XPS bands with high (143.6 and 138.7 eV) and low (142.9 and 136.5 eV) binding energy (BE) are assigned to Pb-Br and uncoordinated Pb species [25], respectively. The uncoordinated Pb species indicate the existence of bromide vacancy. These side peaks gradually decrease from I state to F state, verifying the decreased density of bromide vacancy during light soaking. Moreover, Pb-Br has a higher BE than Cs-Br [26]. The shift of Br 3d XPS peak to higher BE corresponds to the elimination of bromide vacancy [27, 28], which is also observed in the XPS from I state to F state. Therefore, the XPS results confirm the existence of initial defects and the light soaking induced repairing effect.


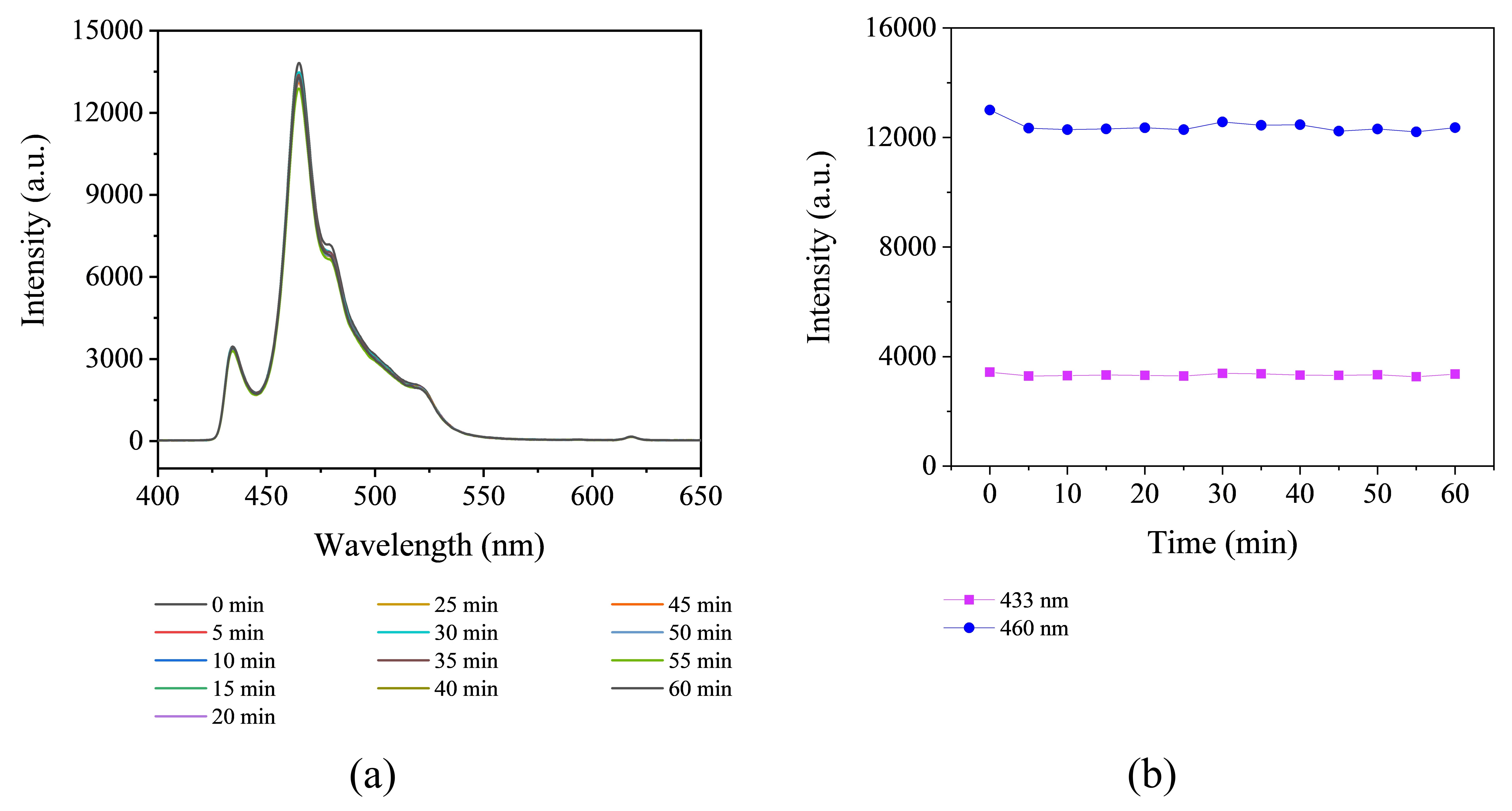


**Figure S13** (a) PL spectra of the I-state Eu-MOFs/perovskites composites excited at 365 nm during light-soaking in vacuum. (b) Intensity of the 460 nm PL peak at different light-soaking time corresponding to (a).


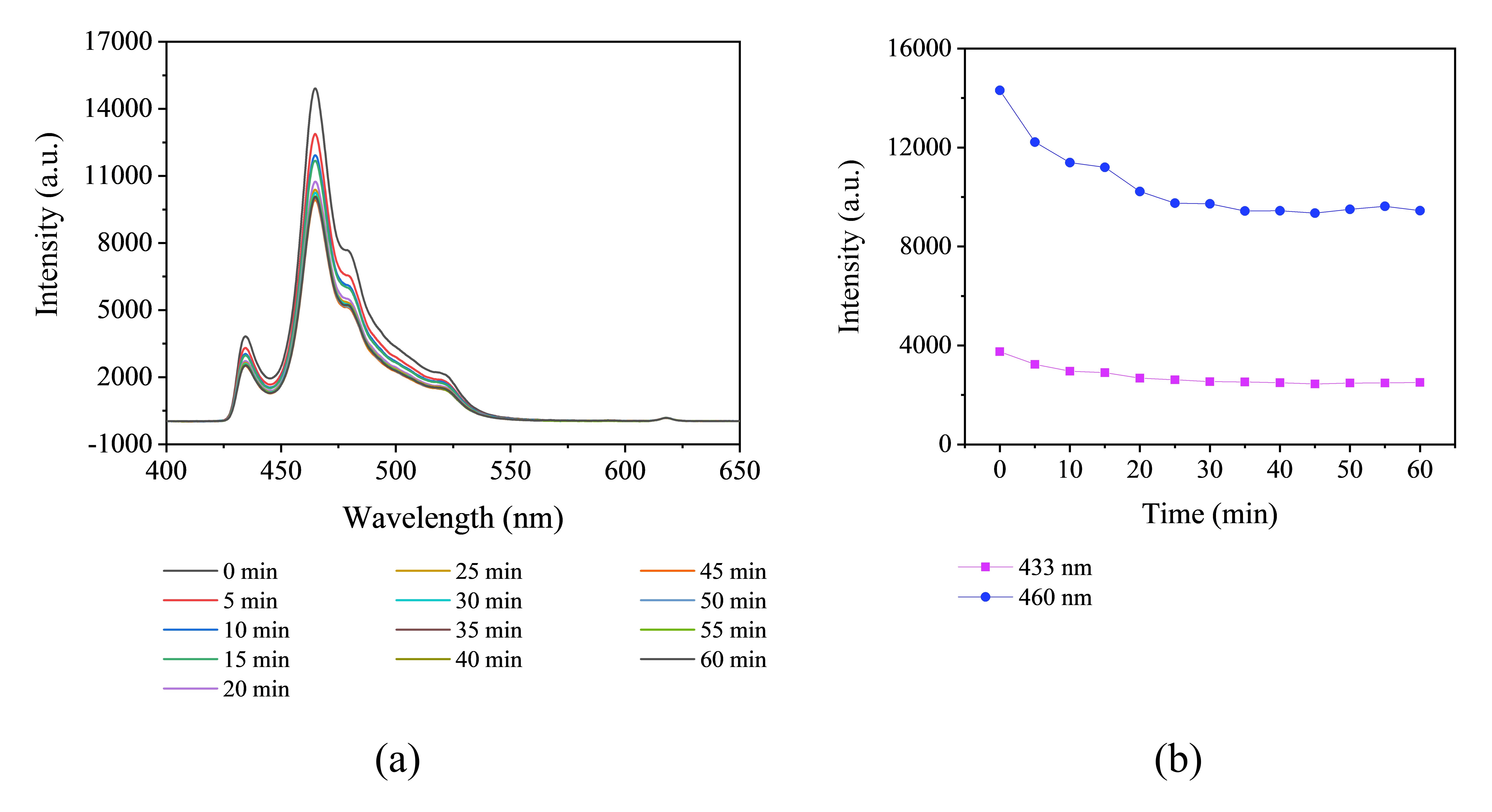


**Figure S14** (a) PL spectra of the I-state Eu-MOFs/perovskites composites excited at 365 nm during continuous heating at 100 ℃. (b) Peak intensities at different heating times, 433 nm (purple line) and 460 nm (blue line).


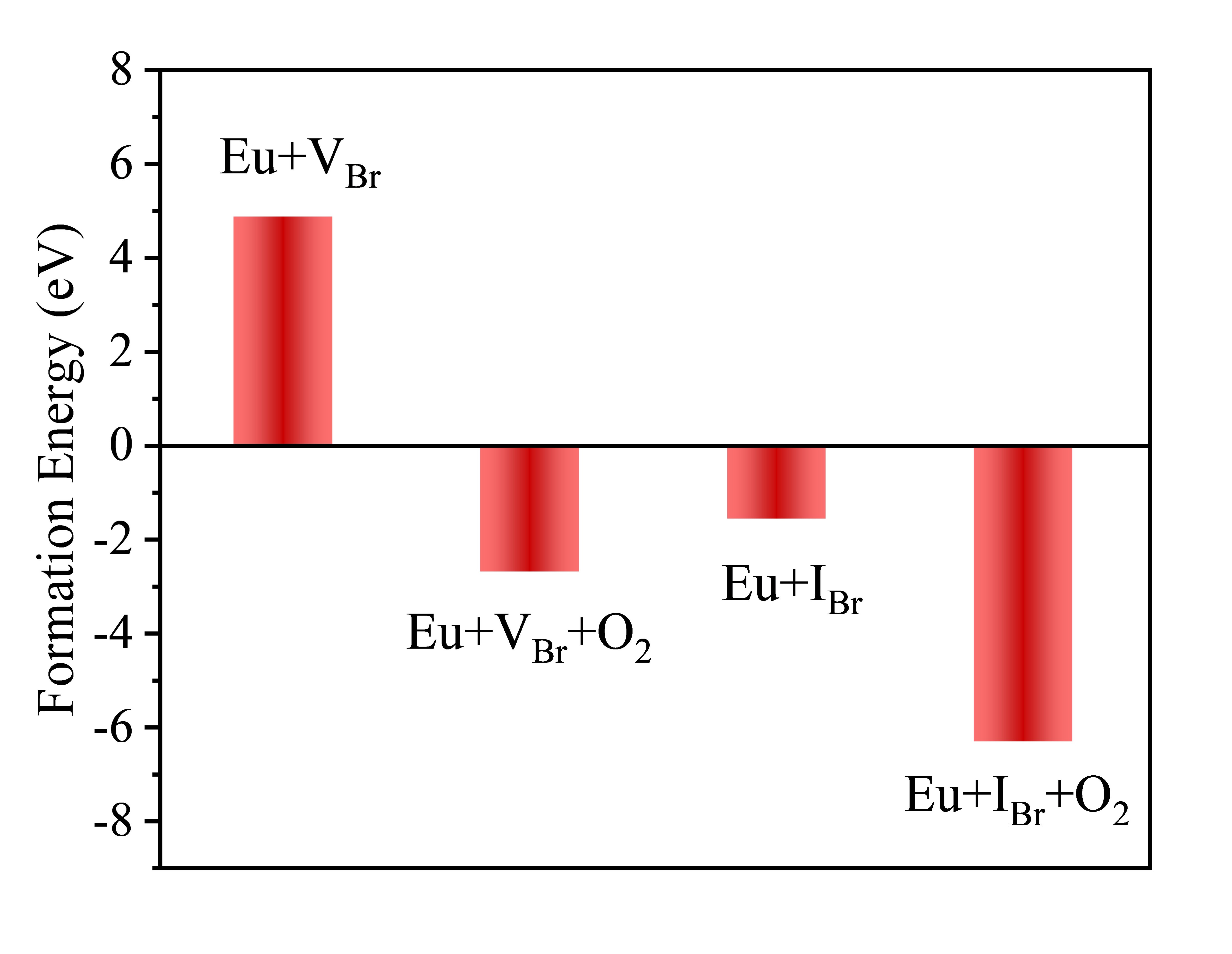


**Figure S15** Formation energies of two different configuations of defects before and after adsorption of O_2_.

The formulas used for formation energy ($E_{f}$) calculations are presented below:

$$E_{f\left( Eu+V_{Br} \right)}=E_{tot\left( Eu+V_{Br} \right)}+E_{Br}-E_{tot(Eu/Pb)}$$

$$E_{f(Eu+V_{Br}+O)}=E_{tot\left( Eu+V_{Br}+O \right)}+E_{Br}-E_{tot(Eu/Pb)}-E_{O}$$

$$E_{f\left( Eu+I_{Br} \right)}=E_{tot\left( Eu+I_{Br} \right)}-E_{Br}-E_{tot(Eu/Pb)}$$

$$E_{f(Eu+I_{Br}+O)}=E_{tot\left( Eu+I_{Br}+O \right)}-E_{Br}-E_{tot(Eu/Pb)}-E_{O}$$

Here, $E_{tot(Eu/Pb)}$ is the total energy of a supercell containing the substitutional dopant Eu replacing Pb. $E_{tot\left( Eu+V_{Br} \right)}$ and $E_{tot\left( Eu+V_{Br}+O \right)}$ are the total energies of the bulk supercell with doping Eu+Br vacancy before and after oxidation, respectively. $E_{tot\left( Eu+I_{Br} \right)}$and $E_{tot\left( Eu+I_{Br}+O \right)}$are the total energies of the bulk supercell with doping Eu+interstitial Br before and after oxidation, respectively. Moreover, $E_{Br}$ and $E_{O}$ are the energies of elements Br and O, respectively.[29] According to the above formulas, negative formation energy implies the structure is stable. Otherwise, positive formation energy implies the structure is not stable. After oxygen doping the formation energy decreases, suggesting both the $Eu+V_{Br}$ and $Eu+I_{Br}$ structures tend to adsorb oxygen.

**

**

**Figure S16** (a) Structural diagram of Cs_4_PbBr_6_ with Eu+V_Br_ after O_2_ adsorption. (b) The calculated PDOS of Cs_4_PbBr_6_ with Eu+V_Br_ after O_2_ adsorption. (c) Structural diagram of Cs_4_PbBr_6_ with Eu+I_Br_ after O_2_ adsorption. (d) The calculated PDOS of Cs_4_PbBr_6_ with Eu+I_Br_ after O_2_ adsorption.


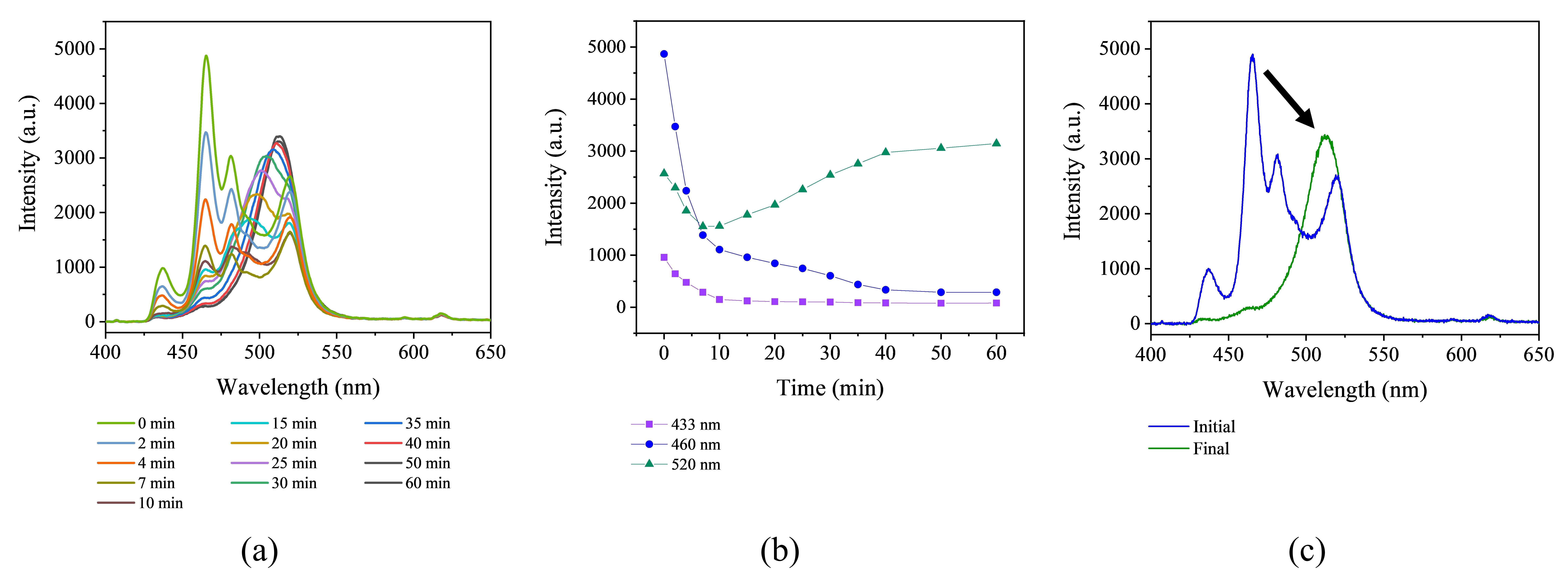


**Figure S17** (a) PL spectra of the I-state Eu-MOFs/perovskites composites excited at 365 nm during light-soaking in a dry environment. (b) PL peak intensities at different irradiation times, 433 nm (purple line), 460 nm (blue line) and 520 nm (green line). (c) The initial PL spectrum before light soaking and the final PL spectrum after light soaking.

As shown in Figure S17, when the light-soaking experiment is conducted in a dry environment, fluorescence variation from blue to green fluorescence is still observed due to the defect repairing process. However, after continuous light soaking for more than one hour, the resulting green emission is yet weaker than the blue emission of the pristine composites. It should be noted that for the comparative experiment conducted at the ambient condition, the final green emission is typically fourfold higher than the initial blue emission.


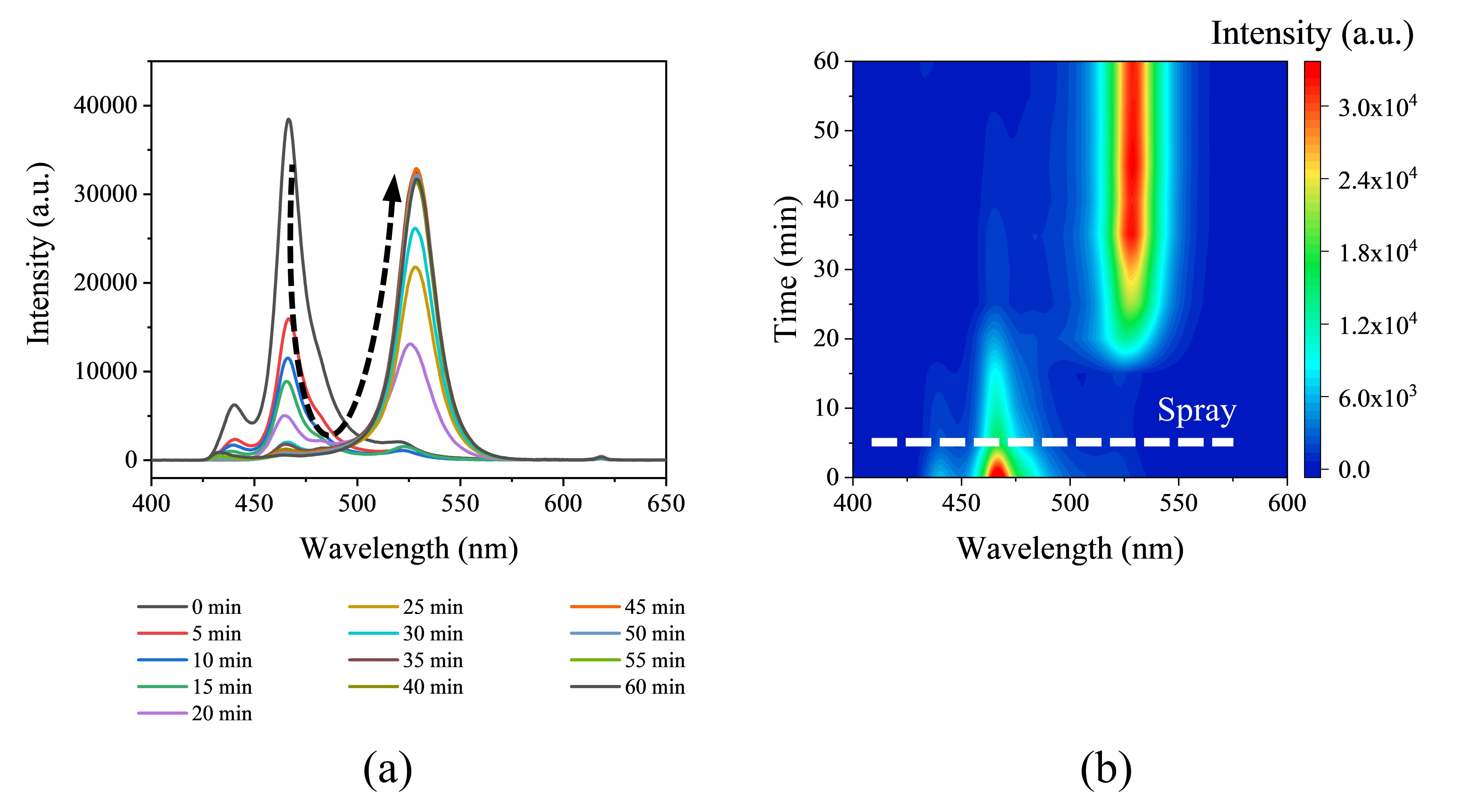


**Figure S18** (a) PL spectra of the I-state Eu-MOFs/perovskites composites excited at 365 nm before and after spray treatment. (b) Pseudocolor contour mapping corresponding to (a).

To further evaluate the role of moisture, the I-state composites were directly treated by spray. As shown in Figure S18, the quenching of blue fluorescence and rising of green fluorescence are observed without light soaking.


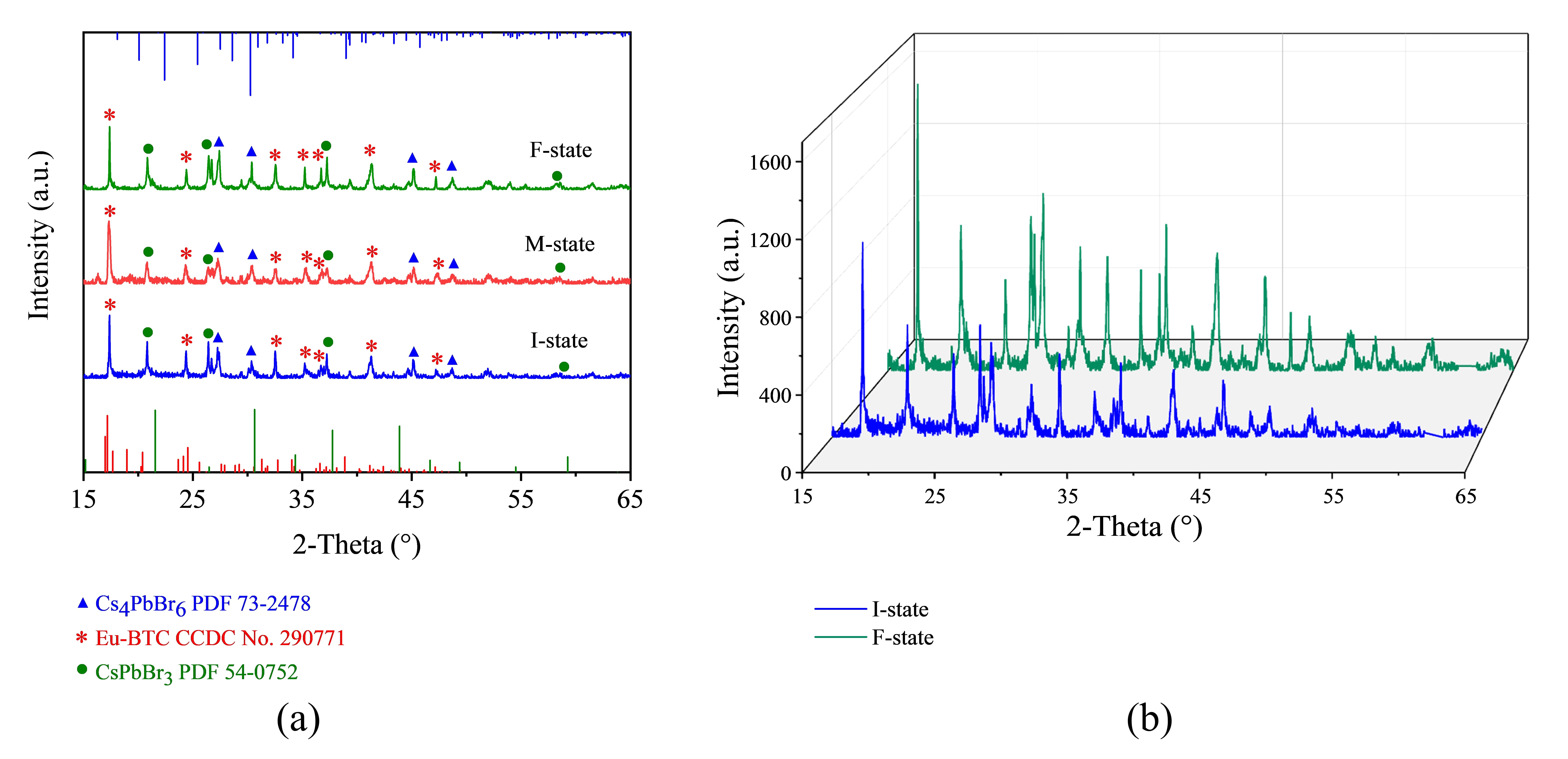


**Figure S19** (a) Normalized XRD patterns of the I-state (black line), M-state (red line) and F-state (blue line) Eu-MOFs/perovskites composites. (b) Comparison of the unnormalized XRD patterns of the I-state and F-state Eu-MOFs/perovskites composites.

**

**

**Figure S20** (a-c) HRTEM images of the Eu-MOFs/perovskites composites at I-state. (d-e) HRTEM images of the Eu-MOFs/perovskites composites at F-state. The blue and green parts highlight 0D Cs_4_PbBr_6_ and 3D CsPbBr_3_ nanodomains, respectively.


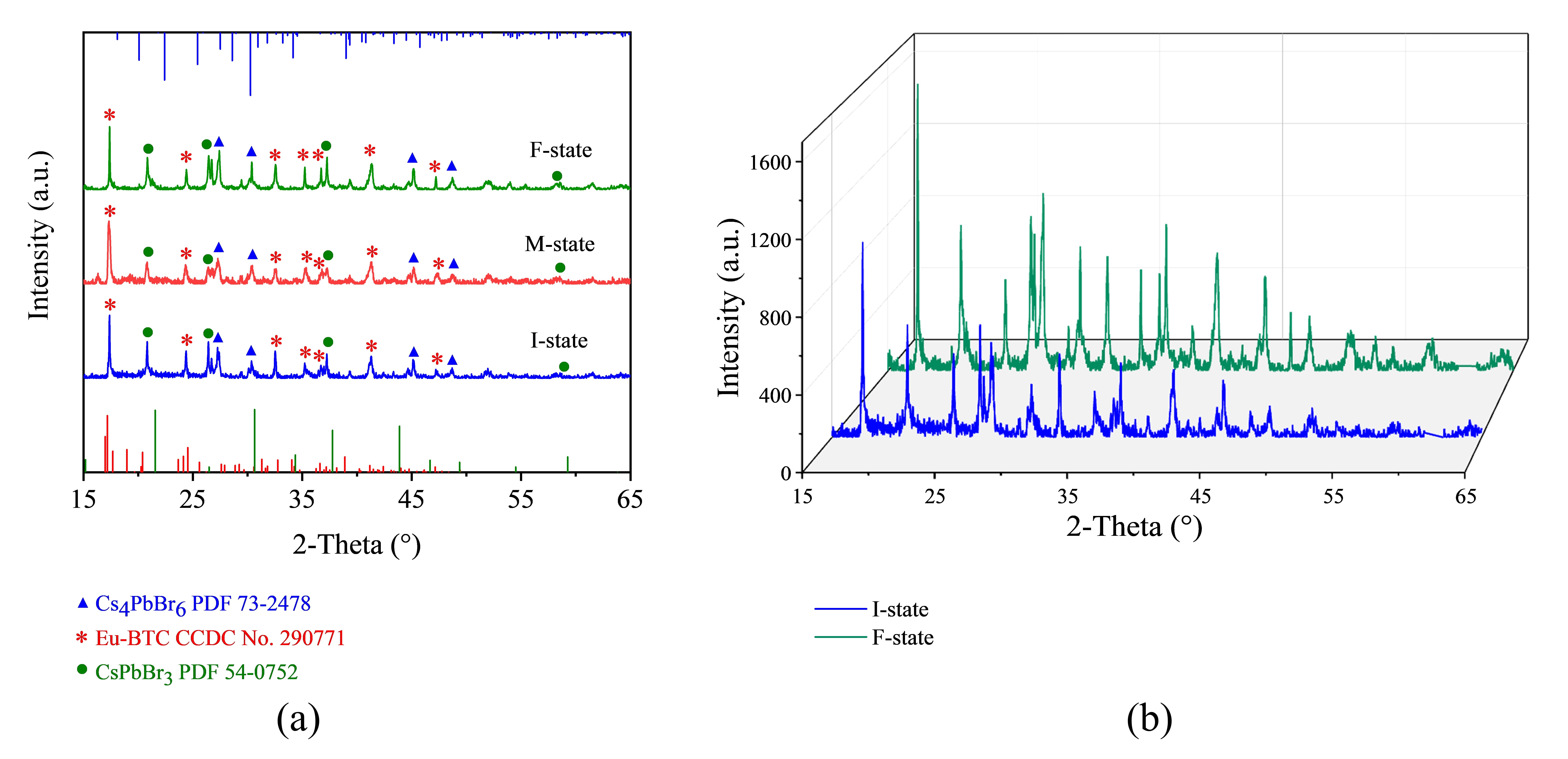


**Figure S21.** Gaussian fitting on the PL spectrum of the pristine Eu-MOFs/perovskites composites excited at 365 nm.

**Table S5** Performance parameters of the LED modules.

**
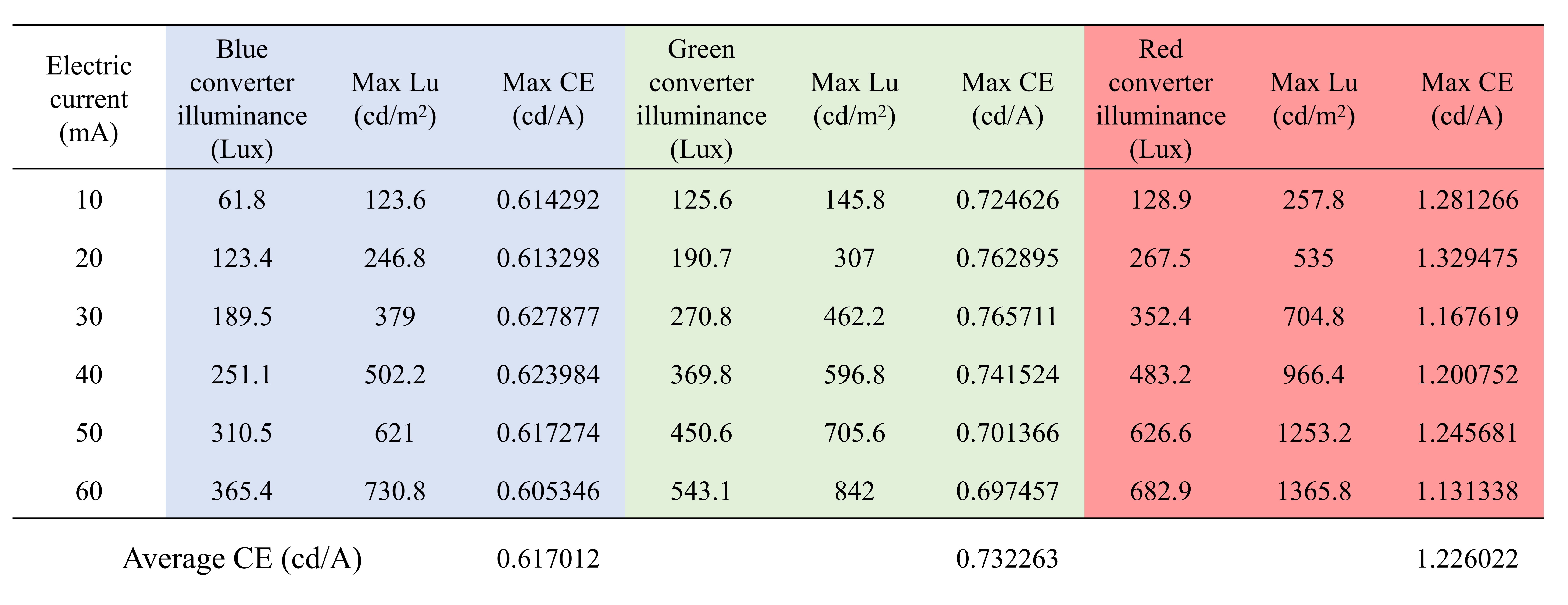
**

**Supplementary information References**

[1] X. Chen, F. Zhang, Y. Ge et al., "Centimeter-sized Cs_4_PbBr_6_ crystals with embedded CsPbBr_3_ nanocrystals showing superior photoluminescence: Nonstoichiometry induced transformation and light-emitting applications," *Advanced Functional Materials*, vol. 28, no. 16, 2018.

[2] X. Wang, Y. Liu, N. Liu et al., "Revisiting the nanocrystal formation process of zero-dimensional perovskite," *Journal of Materials Chemistry A***,** vol. 9, no. 8, pp. 4658-4663, 2021.

[3] R. Sun, N. Liu, W. Zheng et al., "Real-time tracking of emitter generation in a zero-dimensional perovskite," *Chemistry of Materials*, vol. 33, no. 10, pp. 3721-3728, 2021.

[4] H. Shi, Q. Zhang, P. Shi, and X. Zhang, "Full color emission of all-bromide inorganic perovskite nanocrystals," *Applied Physics Letters*, vol. 117, no. 26, 2020.

[5] Q. A. Akkerman, S. Park, E. Radicchi et al., "Nearly monodisperse insulator Cs_4_PbX_6_ (X = Cl, Br, I) nanocrystals, their mixed halide compositions, and their transformation into CsPbX_3_ nanocrystals," *Nano Letters*, vol. 17, no. 3, pp. 1924-1930, 2017.

[6] Q. A. Akkerman, G. Raino, M. V. Kovalenko, and L. Manna, "Genesis, challenges and opportunities for colloidal lead halide perovskite nanocrystals," *Nature Materials*, vol. 17, no. 5, pp. 394-405, 2018.

[7] L. Z. Wu, H. C. Hu, Y. Xu et al., "From nonluminescent Cs_4_PbX_6_ (X = Cl, Br, I) nanocrystals to highly luminescent CsPbX_3_ nanocrystals: Water-triggered transformation through a Csx-stripping mechanism," *Nano Letters*, vol. 17, no. 9, pp. 5799-5804, 2017.

[8] S. Zou, C. Liu, R. Li et al., "From nonluminescent to blue-emitting Cs_4_PbX_6_ nanocrystals: Tailoring the insulator bandgap of 0D perovskite through sn cation doping," *Advanced Materials*, vol. 31, no. 24, 2019.

[9] D. Meggiolaro, E. Mosconi, and F. De Angelis, "Modeling the interaction of molecular iodine with MAPbI_3_: A probe of lead-halide perovskites defect chemistry," *Acs Energy Letters*, vol. 3, no. 2, pp. 447-451, 2018.

[10] D. Meggiolaro, D. Ricciarelli, A. A. Alasmari et al., "Tin versus lead redox chemistry modulates charge trapping and self-doping in tin/lead iodide perovskites," *Journal of Physical Chemistry Letters*, vol. 11, no. 9, pp. 3546-3556, 2020.

[11] P. Li, F. Hussain, P. Cui et al., "Boosting ionic conductivity in antiperovskite Li_3_OCl via defect engineering: Interstitial versus vacancy," *Physical Review Materials*, vol. 3, no. 11, pp. 2019.

[12] J. Yin, Y. H. Zhang, A. Bruno et al., "Intrinsic lead ion emissions in zero-dimensional Cs_4_PbBr_6_ nanocrystals," *Acs Energy Letters*, vol. 2, no. 12, pp. 2805-2811, 2017.

[13] J. Lei, L. Y. Chang, Z. H. Dong, and L. J. Liu, "The role of EuBr_2_ in modulating the crystallization and luminescence of caesium lead bromide," *Materials Research Bulletin*, vol. 137, 2021.

[14] Y. M. Duan, P. P. Li, Y. Lu et al., "Blue-green tunable luminescence mechanism of a novel Tb^3+^ doped Cs_4_PbBr_6_ quantum dots tellurite glass," *Journal of Luminescence*, vol. 231, 2021.

[15] Y. Zhang, M. I. Saidaminov, I. Dursun et al., "Zero-dimensional Cs_4_PbBr_6_ perovskite nanocrystals," *Journal of Physical Chemistry Letters*, vol. 8, no. 5, pp. 961-965, 2017.

[16] M. V. Kovalenko, L. Protesescu, and M. I. Bodnarchuk, "Properties and potential optoelectronic applications of lead halide perovskite nanocrystals," *Science*, vol. 358, no. 6364, pp. 745-750, 2017.

[17] J. Feng, Z. Yang, D. Yang et al., "E-beam evaporated Nb_2_O_5_ as an effective electron transport layer for large flexible perovskite solar cells," *Nano Energy*, vol. 36, pp. 1-8, 2017.

[18] V. Adinolfi, O. Ouellette, M. I. Saidaminov et al., "Fast and sensitive solution-processed visible-blind perovskite uv photodetectors," *Advanced Materials*, vol. 28, no. 33, pp. 7264, 2016.

[19] I. Lignos, R. M. Maceiczyk, M. V. Kovalenko, and S. Stavrakis, "Tracking the fluorescence lifetimes of cesium lead halide perovskite nanocrystals during their synthesis using a fully automated optofluidic platform," *Chemistry of Materials*, vol. 32, no. 1, pp. 27-37, 2019.

[20] V. S. Chirvony, S. González-Carrero, I. Suárez et al., "Delayed luminescence in lead halide perovskite nanocrystals," *The Journal of Physical Chemistry C*, vol. 121, no. 24, pp. 13381-13390, 2017.

[21] F. T. Rabouw, M. Kamp, R. J. van Dijk-Moes et al., "Delayed exciton emission and its relation to blinking in cdse quantum dots," *Nano Letters*, vol. 15, no. 11, pp. 7718-25, 2015.

[22] F. T. Rabouw, J. C. van der Bok, P. Spinicelli et al., "Temporary charge carrier separation dominates the photoluminescence decay dynamics of colloidal cdse nanoplatelets," *Nano Letters*, vol. 16, no. 3, pp. 2047-53, 2016.

[23] S. B. Sun, D. Yuan, Y. Xu et al., "Ligand-mediated synthesis of shape-controlled cesium lead halide perovskite nanocrystals via reprecipitation process at room temperature," *Acs Nano*, vol. 10, no. 3, pp. 3648-3657, 2016.

[24] H. T. Chen, A. Q. Guo, J. Zhu et al., "Tunable photoluminescence of CsPbBr_3_ perovskite quantum dots for their physical research," *Applied Surface Science*, vol. 465, pp. 656-664, 2019.

[25] J. Y. Woo, Y. Kim, J. Bae et al., "Highly stable cesium lead halide perovskite nanocrystals through in situ lead halide inorganic passivation," *Chemistry of Materials*, vol. 29, no. 17, pp. 7088-7092, 2017.

[26] V. I. Nefedov, "A comparison of results of an esca study of nonconducting solids using spectrometers of different constructions," *Journal of Electron Spectroscopy and Related Phenomena*, vol. 25, no. 1, pp. 29-47, 1982.

[27] G. Ebbinghaus, and A. Simon, "Electronics structures of Rb, Cs and some of their metallic oxides studied by photoelectron spectroscopy," *Chemical Physics*, vol. 43, no. 1, pp. 117-133, 1979.

[28] W. E. Morgan, W. J. Stec, and J. R. Van Wazer, "Inner-orbital binding-energy shifts of antimony and bismuth compounds," *Inorganic Chemistry*, vol. 12, no. 4, pp. 953-955, 1973.

[29] X. Wang, Y. Liu, N. Liu et al., "Revisiting the nanocrystal formation process of zero-dimensional perovskite," *Journal of Materials Chemistry A*, vol. 9, no. 8, pp. 4658-4663, 2021.
